# A fermented Mistletoe (*Viscum album* L.) extract elicits markers characteristic for immunogenic cell death driven by endoplasmic reticulum stress in vitro

**DOI:** 10.1186/s12906-025-04909-8

**Published:** 2025-05-14

**Authors:** Ulrike Weissenstein, Sibylle Tschumi, Bettina Leonhard, Stephan Baumgartner

**Affiliations:** 1 Society for Cancer Research, Arlesheim, Switzerland; 2 Institute of Integrative Medicine, Witten/Herdecke University, Herdecke, Germany

**Keywords:** Mistletoe, Viscum album, Cancer, Immunogenic cell death, Danger associated molecular patterns, Endoplasmic reticulum stress, Reactive oxygen species

## Abstract

**Background:**

Immune evasion is a characteristic hallmark of cancer. Immunotherapies aim to activate and support the body's immune system to recognize and fight tumor cells. Induction of immunogenic cell death (ICD) and the associated activation of danger signaling pathways can increase the immunogenicity of tumor cells. Therapeutic ICD stimuli activate endoplasmic reticulum stress pathways and apoptosis leading to the cellular expression of damage-associated molecular patterns (DAMPs).

The aim of our in vitro study was to investigate whether mistletoe extracts induce characteristics of immunogenic tumor cell death in cancer cell lines.

**Methods:**

Three human breast cancer cell lines and one murine melanoma cell line (SKBR3, MDA-MB-231, MCF-7, and B16F10) were treated with aqueous, fermented *Viscum album* extract (VAE: Iscador Qu spec.) and taxol or tunicamycin as positive controls, respectively. To investigate whether VAE induces ribotoxic stress, we measured the ER stress regulators p-eIF2a, ATF4, and CHOP by Western blot. Cell surface exposure of DAMPs (calreticulin, heat shock proteins hsp70 and hsp90), apoptosis and induction of mitochondrial reactive oxygen species (ROS) were assessed by flow cytometry. HMGB1 and ATP were quantified by ELISA and chemiluminescence assay, respectively.

**Results:**

Treatment with VAE resulted in phosphorylation of eIF2α in all cancer cell lines tested and increased calreticulin (CRT) exposure on the surface of pre-apoptotic SKBR3 breast cancer and B16F10 mouse melanoma cells. VAE exerted a concentration-dependent effect in all cell lines, resulting in a significantly increased exposure of three DAMPs (CRT, hsp70 and hsp90) on the surface of early apoptotic cells. Furthermore, VAE elevated mitochondrial ROS production and the release of ATP. HMGB1 release was not induced by VAE.

**Conclusions:**

In this in vitro study, we demonstrated for the first time the potential of a mistletoe extract to induce surrogate markers of immunogenic cancer cell death. This is a primary step in investigating the potential of VAEs to contribute to ICD-induced tumor-specific immune activation.

**Supplementary Information:**

The online version contains supplementary material available at 10.1186/s12906-025-04909-8.

## Background

Tumors use various escape mechanisms to prevent the patient's immune system from recognizing and eliminating them [1, 2]. In recent years, the immunological approach to cancer treatment has come to the focus of attention with the aim of breaking the immunological tolerance to tumor cells. Particular attention has been paid to the induction of immunogenic cancer cell death, which is an important step in initiating a specific anti-cancer T cell response. Triggering an immunogenic cancer cell death can make an existing tumor recognizable and attackable again for the immune system [3].

Several factors are responsible for the immunogenicity of cell death. These include the intrinsic antigenicity of the cells, the induction of stress responses prior to cell death, the nature of the cytotoxic reagent, the death pathway involved, and the presence of immune cells capable of responding. An ideal inducer of immunogenic cell death (ICD) activates different pathways such as apoptotic cell death, endoplasmic reticulum (ER) stress response, induction of reactive oxygen species (ROS), autophagy, necroptosis or secondary necrosis. Activation of these pathways leads to the expression of damage-associated molecular patterns (DAMPs) such as phosphatidylserine, chaperones such as calreticulin, heat shock proteins (e.g. hsp70, hsp90), HMGB1, ATP and others on the cell surface of or secreted by dying cells [3–5].

When the endoplasmic reticulum is stressed, proteins accumulate due to improper folding, modulation, or trafficking. As a result, three stress sensor proteins (IRE1, PERK, ATF6) initiate the unfolded protein response (UPR) signaling pathway [6]. The protein kinase R (PKR)-like endoplasmic reticulum kinase (PERK) arm regulates the translational response of the UPR. PERK is a transmembrane protein with a stress-sensing domain and a protein kinase domain. Upon ER stress, PERK dimerizes and promotes its autophosphorylation and activation. Activated PERK phosphorylates eukaryotic translation initiation factor 2 A (eIF2α), resulting in a transient slowing of the overall rate of translation initiation, the prevention of further protein synthesis [7], and the induction of activating transcription factor 4 (ATF4). ATF4 contributes to the increased folding capacity of ER proteins.

The main goal of UPR is to restore protein homeostasis to ensure cell survival. However, under prolonged and severe ER stress and sustained PERK activation, a signaling switch occurs that leads to apoptosis and cell death. The activated transcription factor C/EBP-homologous protein (CHOP) is involved in the regulation of apoptosis [8].

The induction of ER stress triggers danger signaling pathways leading to the release of danger signals and apoptosis [9, 10]. Ideally, the release of DAMPs, danger signals released by injured cells or tissues, into the tumor microenvironment initiates the so-called cancer-immunity cycle [11]. DAMPs attract and activate dendritic cells (DCs), which in turn engulf and digest tumor cell fragments. Activated DCs transport tumor cell-specific antigens to the lymph nodes, where they encounter and activate naive specific T cells, followed by their clonal expansion and dissemination throughout the body. When the activated tumor-specific cytotoxic T cells encounter the tumor cells, they can attack and eliminate them. In addition, a systemic protective anti-tumor immunity, the so-called abscopal immune response, is possible [12].

The first step in the cancer immunity cycle is the induction of immunogenic tumor cell death, which leads to the release of tumor cell-specific and/or tumor-associated antigens and DAMPs. Known inducers of immunogenic cell death include chemotherapeutics such as anthracyclines, cyclophosphamide, certain endocrine and targeted therapies, photodynamic and radiation therapy [13, 14]. ICD inducers fit well into the concept of intratumoral immunotherapy. Local application of immunostimulatory reagents into the tumor tissue aims to induce a strong antitumor immune response using the tumor as its own vaccine. In combination with systemic immunotherapies, this could lead to an improved response rate and better tolerability of the treatment [15, 16].

European mistletoe (*Viscum album* L.) belongs to the Santalaceae family and is an evergreen, semi-parasitic plant. *Viscum album* ssp. *album* grows on a variety of trees, such as *Quercus robur* L. or *Malus domestica*. Mistletoe preparations are prescribed to cancer patients as supportive treatment, either alone or in combination with conventional oncological therapies. Aqueous mistletoe preparations contain various bioactive compounds such as mistletoe lectins (MLs), viscotoxins, and polysaccharides. These components, especially the MLs and viscotoxins, have been chemically and pharmacologically characterized in numerous studies [17–21]. Urech et al. specifically investigated the MLs and viscotoxins in the fermented mistletoe preparation we used [22]. Some of the known antitumor effects of mistletoe preparations are immunomodulatory activities, induction of apoptosis, and cell cycle arrest [23–25].

The aim of our study was to investigate whether mistletoe extracts induce cancer cell death with molecular features of immunogenic cell death in vitro. This is a first step in exploring the potential of mistletoe extracts to contribute to the stimulation of an anti-tumor immune response by inducing the release of cell death related immunogenic signals.

## Methods

### Extracts and reagents

The aqueous, fermented mistletoe preparation Iscador Qu spec. 20 mg (VAE, from the host trees *Quercus robur* and *Q. petraea*, lot 9019/70, expiration date 07–2021: total mistletoe lectin concentration 1689 ng/mL and lot 1021/70, expiration date 07–2023: total mistletoe lectin concentration 1592 ng/mL) was obtained from Iscador AG (Arlesheim, Switzerland). To prepare Iscador, mistletoe leaves, stems and berries are harvested in mid-summer and mid-winter. The fresh plant parts are ground and fermented for 3 days after the addition of *Lactobacillus plantarum*. The solution is separated from the solid material and the aqueous summer and winter extracts obtained are subsequently mixed. Iscador is authorized by Swissmedic, the Swiss regulatory authority for medicines and medical devices. The detailed manufacturing process of the extract is described in the Anthroposophic Pharmaceutical Codex of the International Association of Anthroposophic Pharmacists [26].

Taxol (#T7402), tunicamycin (#T7765) and mitoxantrone dihydrochloride (#M6545) were purchased from Sigma-Aldrich GmbH/Merck & Cie (Buchs, CH).

### Cell lines and cell culture

The human breast carcinoma cell lines SKBR3 (RRID:CVCL_0033), MCF-7 (RRID:CVCL_0031), and MDA-MB-231 (RRID:CVCL_0062) (obtained from DSMZ, German Collection of Microorganisms and Cell Cultures, Braunschweig, Germany) and the mouse melanoma cell line B16F10 (RRID:CVCL_0159) (cells kindly provided by Prof. A. Zippelius, University of Basel, Switzerland) were used for the experiments.

The cell lines were grown in the following media:

- SKBR3 in McCoy’s 5A (Sigma-Aldrich), 10% fetal calf serum, 1% penicillin/streptomycin, 2 mM L-glutamine (Sigma-Aldrich)
- MDA-MB-231 in DMEM (hg) medium, 10% fetal calf serum and 1% penicillin/streptomycin (Sigma-Aldrich)
- MCF-7 in DMEM (hg) medium, 10% fetal calf serum, 1% penicillin/streptomycin, 1 mM sodium pyruvate, and 10 μg/mL insulin (Sigma-Aldrich)
- B16F10 in DMEM (hg) medium, 10% fetal calf serum, 1% penicillin/streptomycin, 1 mM sodium pyruvate

Cells were grown exponentially in a humidified atmosphere of 5% CO_2_ at 37 °C and harvested from subconfluent monolayers using trypsin–EDTA (Sigma-Aldrich).

### Drug concentrations in the assays

For each of the different cell lines, we selected the concentrations of VAE and the reagents used as positive controls at which we could detect concentration-dependent apoptosis as well as the expression of DAMPs. The preliminary dose–response data are provided in Supplementary Material 3.

### Western blot analysis

For Western blotting, cells were treated with experimental or control medium for 24 h in 6-well cell culture dishes. Total protein was extracted using RIPA Lysis and Extraction buffer (#89900, Thermo Fisher Scientific) in the presence of phosphatase inhibitors (#78440, Thermo Fisher Scientific). Protein concentration was determined by the Bradford assay using bovine serum albumin (Sigma Aldrich) as a protein standard.

Proteins were separated by SDS–polyacrylamide gel electrophoresis using the BioRad system with 4–20% MP-TGX stain-free polyacrylamide gels (stain-free activation of total protein for 1 min in the UV box of a Vilber Fusion Solo S imaging system) (BioRad). Proteins were transferred to PVDF membranes using the Trans-Blot Turbo system (BioRad) and incubated with primary antibodies (recombinant anti-EIF2S1 (phospho S51) antibody [E90] Abcam Cat# ab32157, RRID: AB_732117); recombinant anti-ATF-4 antibody [EPR18111], (Abcam Cat# ab184909, RRID: AB_2819059); anti-DDIT3 antibody [9 C8], (Abcam Cat# ab11419, RRID: AB_298023) overnight at 4 °C and then with goat-anti-rabbit IgG H&L (HRP) (Abcam Cat# AB205718) for 1 h at room temperature, followed by Clarity Western ECL substrate (Biorad, #1705060) incubation.

### Mitochondrial superoxide production assay

Mitochondrial superoxide production was measured using the MitoSOX Red Superoxide Indicator (ThermoFisher Scientific, #M36008). The MitoSOX™ Red Mitochondrial Superoxide Indicator is a fluorogenic dye for the selective detection of superoxide in the mitochondria of living cells.

500′000 cells/well were seeded and grown in 6-well plates in DMEM low glucose medium supplemented with 2% fetal calf serum and 1% penicillin/streptomycin (Sigma-Aldrich). After treatment with VAE for 24 h, cells were trypsinized, transferred to 5 mL Facstubes, washed, and suspended in 0.5 mL prewarmed assay buffer (HBSS with calcium and magnesium, Sigma-Aldrich). Depending on the cell line, 30–60 µM MitoPQ, mitochondria-targeted redox cycler (Abcam plc, Cambridge, UK, Cat# ab146819) was used as a positive control. After the addition of 1 µM MitoSOX, cells were incubated for 30 min in a shaking water bath at 37 °C, protected from light. DMSO was used as a vehicle control. Cells were washed three times and resuspended in 0.5 mL assay buffer. After the addition of DAPI to exclude dead cells, samples were immediately analyzed using a FACSCanto II (BD Biosciences, San Jose, CA). Values are expressed as MFI (geometric mean).

### Flow cytometry analysis

DAMP expression on the surface of non-permeabilized cells and apoptosis were determined by flow cytometry. After treatment with VAE or taxol or tunicamycin in 6-well cell culture plates for 24 or 48 h, cells were trypsinized, washed, and suspended in PBS containing 0.5% FCS. Cells were co-stained with either recombinant PE anti-calreticulin antibody [EPR3924]—ER marker (Abcam Cat# ab209577, RRID: AB_2943490) and Alexa Fluor® 488 anti-Hsp70 antibody (BioLegend Cat# 648004, RRID: AB_2119537) or with recombinant Alexa Fluor® 488 anti-Hsp90 antibody [EPR16621-67] (Abcam Cat# ab223467, RRID: AB_2943491) for 30 min on ice in the dark. After washing with Annexin V Binding Buffer (BD Biosciences), cells were incubated with APC Annexin V (#550475, BD Biosciences) or with either 7-AAD or DAPI (#559925/#564907, BD Biosciences) for 15 min at 4 °C in the dark to monitor apoptosis and cell death. Samples were immediately analyzed using a FACSCanto II. Data were analyzed using FlowJo 10.6.2 software (Ashland OR, USA). Early apoptotic cells: Annexin V positive and 7-AAD/DAPI negative. Late apoptotic and/or necrotic cells: Annexin V positive and 7-AAD/DAPI positive. Values are expressed as a percentage of total cell number. Cells were interpreted as pre-apoptotic if they belonged to the population of Annexin V- and 7-AAD/DAPI-negative cells whose mean fluorescence intensity (MFI, geometric mean) for calreticulin was significantly higher than that of untreated control cells.

### HMGB1 assay

Cells were seeded, treated with VAE, taxol or mitoxantrone dihydrochloride (Sigma-Aldrich, #M6545) (positive control), and cell culture supernatants were collected at 24 and 48 h and frozen at −80 °C. HMGB1 levels in cell supernatants were quantified using a commercial ELISA kit (Elabscience Biotech, #E-EL-H1554) according to the manufacturer's instructions. The detection limit was 25.31 pg/mL.

### ATP assay

Cells were seeded, treated with VAE or taxol, and cell culture supernatants were collected at 6 and 24 h and frozen at −80 °C. The extracellular ATP concentration in cell supernatants was analyzed using an ATP chemiluminescence assay kit (Molecular Probes, # A22066) according to the manufacturer's instructions.

Briefly, 10 µl each of standards or supernatants were added to 90 µl of reaction solution. After incubation for 15 min, luminescence was measured using a Wallac Victor II plate reader.

### Data analysis

Data were analyzed by either one-way analysis of variance (ANOVA, uncorrected Fisher's LSD), Friedman test (uncorrected Dunn's test), or Spearman nonparametric correlation analysis using GraphPad Prism, version 9.4.0. The significance level was defined as *p* ≤ 0.05.

## Results

### ER stress signaling pathway

**Fig. 1:**
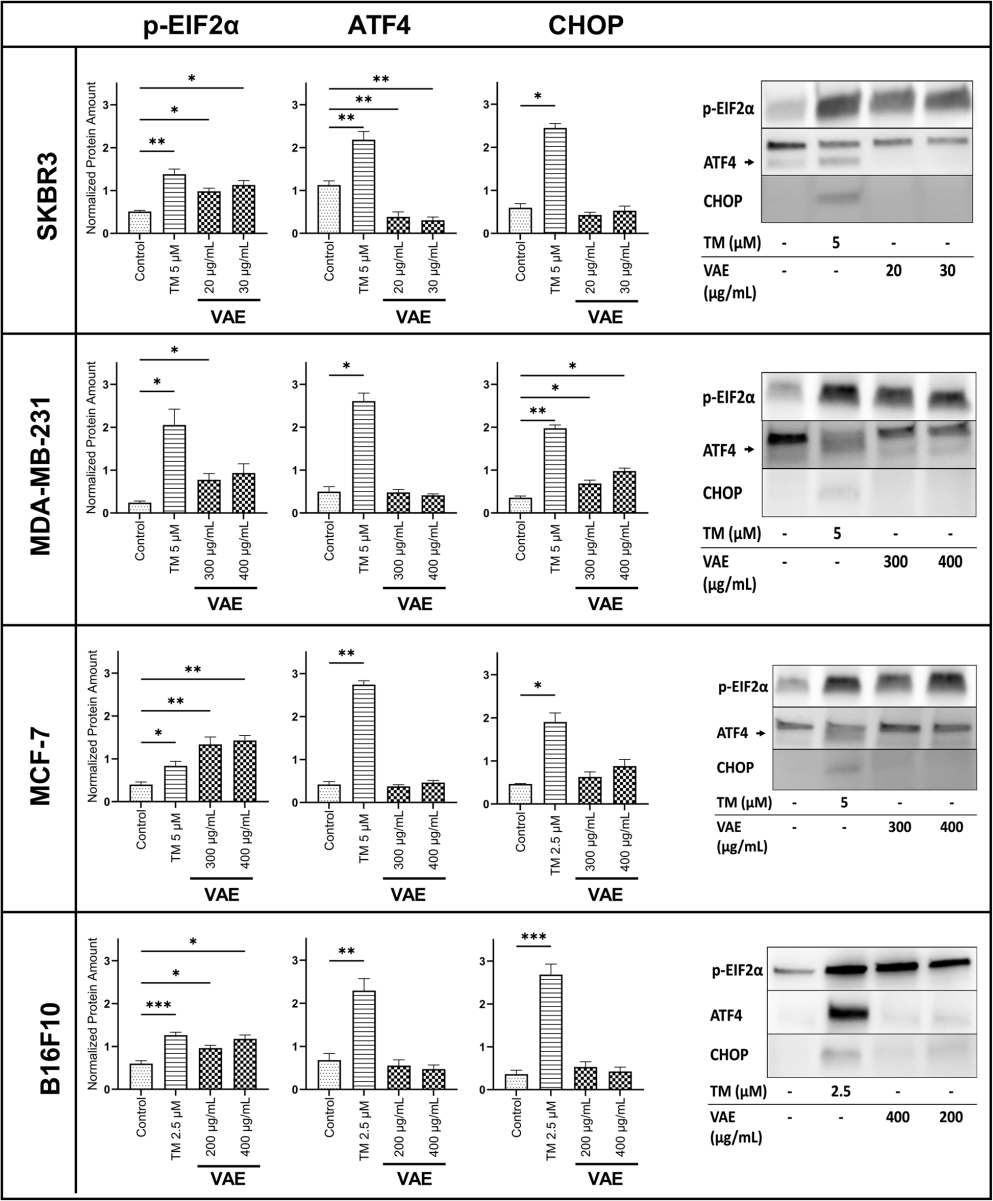
The expression levels of p-eIF2α, ATF4 and CHOP after treatment with indicated reagents for 24 h were determined by stain free Western blot analysis. Normalized data are presented as mean ± SE of at least 3 independent experiments with SKBR3, MDA-MB-231, MCF-7 and B16F10 cells. Representative western blot images are shown for all cell lines. The arrow indicates the location of ATF4 protein bands. Significance values are given relative to untreated controls (*p < 0.05, **p < 0.01, ***p < 0.001)

To investigate whether the aqueous, fermented mistletoe preparation (*Viscum album* extract, VAE) induces ribotoxic stress, we analyzed the ER stress regulators p-eIF2α, ATF4, and CHOP by Western blot. Tunicamycin (TM), a compound routinely used to activate an ER stress response including the ER stress markers mentioned above, was used as a positive control [27, 28]. Both VAE and TM significantly increased eIF2α phosphorylation in all cell lines tested. In addition, the ER stress inducer TM significantly stimulated the expression of ATF4 and CHOP in all cell lines, whereas VAE had no stimulating effect on the expression of ATF4 and CHOP under the experimental conditions used, except for a significant increase of CHOP in the triple negative MDA-MB-231 cells. Treatment with VAE in SKBR3 cells resulted in a significant (*p* < 0.01) reduction of ATF4 (Fig. 1).

As we will also show for the expression of DAMPs, we observed large sensitivity differences between the cell lines regarding the VAE concentrations necessary to induce phosphorylation of the ER stress regulator eIF2α. In SKBR3, a VAE concentration of 20–30 µg/mL was sufficient, whereas 200–400 µg/mL VAE were required for the induction of p-eIF2α in MCF-7, MDA-MB-231 and B16F10 cells (Fig. 1). Full uncropped blot images are shown in Supplementary Material 2.

### Mitochondrial superoxide production

**Fig. 2:**
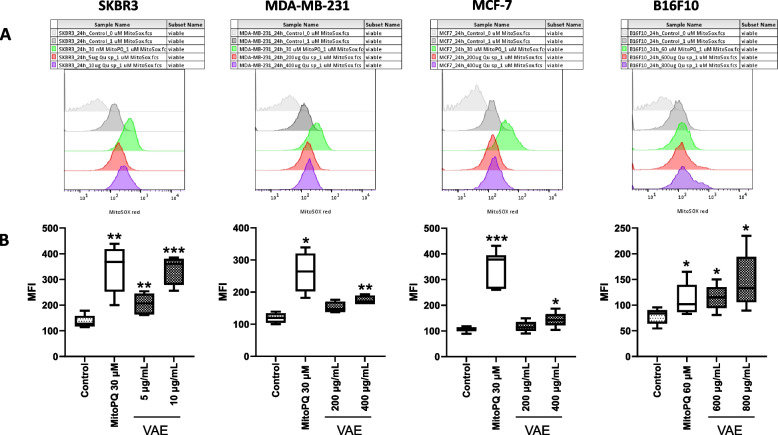
Analysis of mitochondrial superoxide production. SKBR3, MDA-MB-231, MCF-7 and B16 F10 cells were treated with either MitoPQ (positive control) or VAE for 24 h, labelled with MitoSOX red Superoxide Indicator and analyzed by flow cytometry. **A** Representative FACS images of MitoSOX red fluorescence intensity and **B** Box and whiskers plots represent the MFI data (Min to Max) from at least 3 independent experiments. Significance values are given relative to untreated controls (*p < 0.05, **p < 0.01, ***p < 0.001)

We detected mitochondrial superoxide generation using the MitoSOX red (MSR) superoxide indicator, which rapidly and selectively targets the mitochondria. Positive control MitoPQ is a redox cycler that selectively increases mitochondrial superoxide production [29]. In all cell lines, we detected a concentration-dependent, significantly increased mitochondrial superoxide production after 24 h of treatment with either VAE or the positive control MitoPQ. The human SKBR3 cell line was more sensitive to VAE than the other cell lines, and a significant increase in mitochondrial superoxide production was induced by VAE concentrations of 5 and 10 µg/mL, whereas in the other two breast cancer cell lines, 400 µg/mL and in murine B16F10 cells, 600–800 µg/mL VAE was used to achieve a significant effect. The results are shown in Fig. 2.

### DAMP exposure, apoptosis, and cell death

We determined the exposure of the DAMPs calreticulin (CRT), heat shock protein 70 (hsp70), and hsp90 on the cell surface of DAPI/7-AAD negative cells by flow cytometry. The ICD-inducing chemotherapeutic taxol (paclitaxel) [30] and TM were used as positive controls. Taxol has been shown to induce the release of DAMPs such as calreticulin, ATP and HMGB1 in ovarian cancer cells [31].

**Fig. 3:**
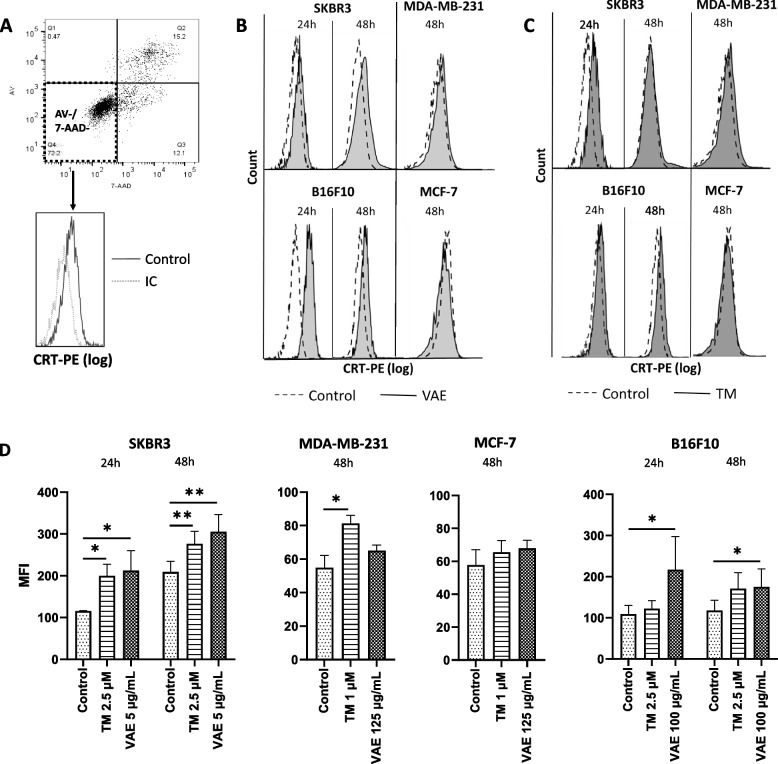
Exposure of calreticulin on the surface of pre-apoptotic (annexin-V-negative and 7-AAD/DAPI-negative) cells. **A** Gating strategy of pre-apoptotic cells and representative FACS histogram plot of CRT exposure on untreated control vs. isotype control. Representative FACS histogram plots of CRT-exposure on pre-apoptotic cells after treatment with either **B** VAE or **C** TM vs. untreated control. **D** FACS analysis of the mean fluorescence intensity (MFI, geomean) of CRT exposure on the surface of cells treated with TM or VAE, respectively. Results are presented as mean ± SE from at least 3 independent experiments. Significance values are given relative to untreated controls (*p < 0.05, **p < 0.01)

Calreticulin exposure on the surface of pre-apoptotic (Annexin-V-negative and 7-AAD/DAPI-negative) cells was highly cell line-dependent and varied significantly (Fig. 3). We observed a significant increase in CRT on the surface of pre-apoptotic SKBR3 cells after treatment with both TM and VAE for 24 h and 48 h. In B16F10 cells, CRT increased significantly after treatment with VAE (24 h and 48 h). TM treatment had no effect. Considering that MDA-MB-231 and MCF-7 cells are much more resistant to treatment with VAE and that in pre-experiments no DAMP exposure was detectable at all after 24 h, we examined these cell lines only after 48 h treatment. In MDA-MB-231 cells, only treatment with TM resulted in significantly increased DAMP exposure, and in MCF-7 cells, none of the treatments induced DAMP exposure on the surface of pre-apoptotic cells.

Because the size of taxol-treated cells increased significantly due to swelling, resulting in a fluorescence shift of the entire cell population, the corresponding MFI data were not included in the analysis.

**Fig. 4:**
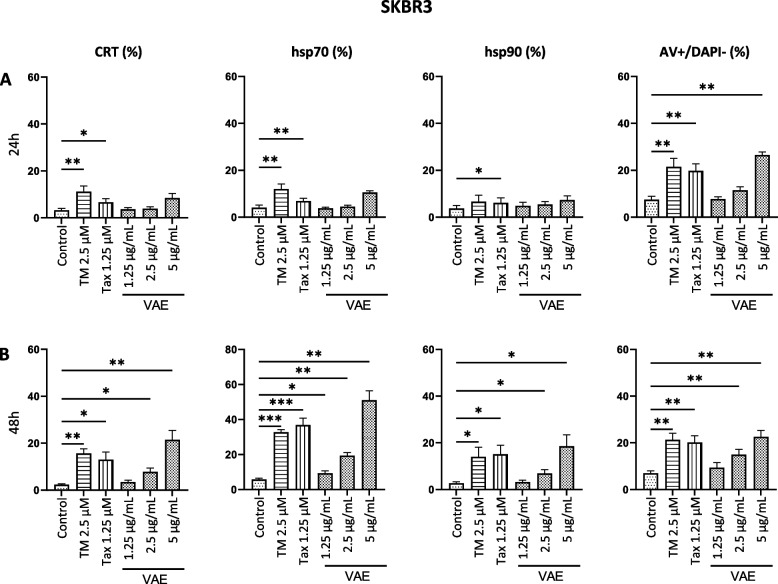
Cell surface exposure of calreticulin (CRT), hsp70, hsp90 or AV +/DAPI- (early apoptotic) cells after treatment of SKBR3 cells for **A**. 24 h or **B**. 48 h with either tunicamycin (TM), taxol (Tax) or viscum album extract (VAE). Results are presented as mean ± SE from at least 3 independent experiments. Significance values are given relative to untreated controls (*p < 0.05, **p < 0.01, ***p < 0.001)

**Fig. 5:**
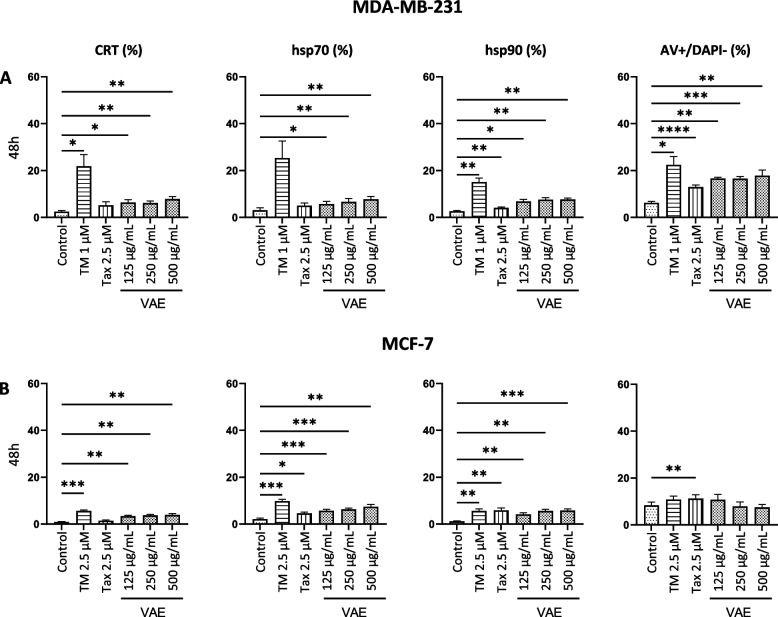
Cell surface exposure of calreticulin (CRT), hsp70, hsp90 or AV +/DAPI- (early apoptotic) cells after treatment of **A**. MDA-MB-231 or **B**. MCF-7 cells for 48 h with either tunicamycin (TM), taxol (Tax) or viscum album extract (VAE). Results are presented as mean ± SE from at least 3 independent experiments. Significance values are given relative to untreated controls (*p < 0.05, **p < 0.01, ***p < 0.001)

**Fig. 6:**
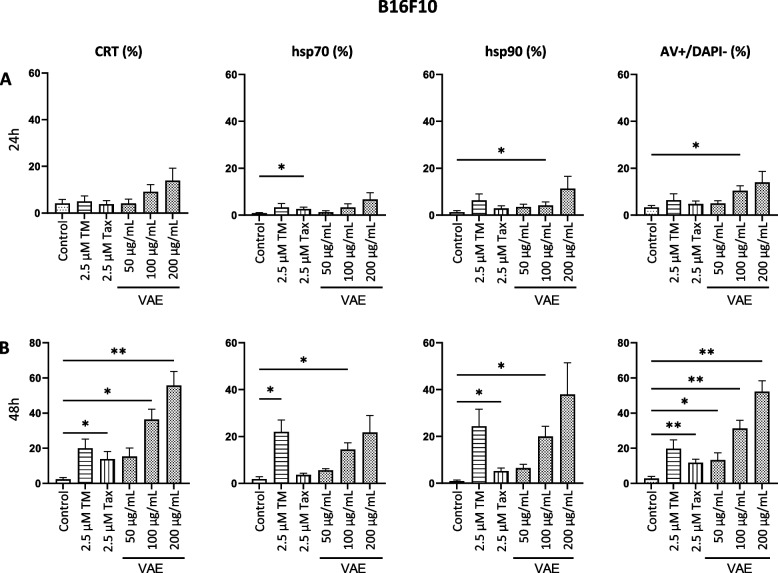
Cell surface exposure of calreticulin (CRT), hsp70, hsp90 or AV +/DAPI- (early apoptotic) cells after treatment of B16F10 cells for **A**. 24 h or **B**. 48 h with either tunicamycin (TM), taxol (Tax) or viscum album extract (VAE). Results are presented as mean ± SE from at least 3 independent experiments. Significance values are given relative to untreated controls (*p < 0.05, **p < 0.01)

We also determined the amount of DAMPs on the surface of 7-AAD/DAPI negative cells in relation to the phosphatidylserine expression (Annexin V positive, early apoptotic cells) (Supplementary Fig. 1, Supplementary Fig. 2). Our results indicate a strong variability in the sensitivity of the individual cell lines to VAE, TM, and taxol. All cell lines exposed the investigated DAMPs on the surface of early apoptotic cells after treatment with either VAE or tunicamycin (Figs. 4, 5 and 6).

**Fig. 7:**
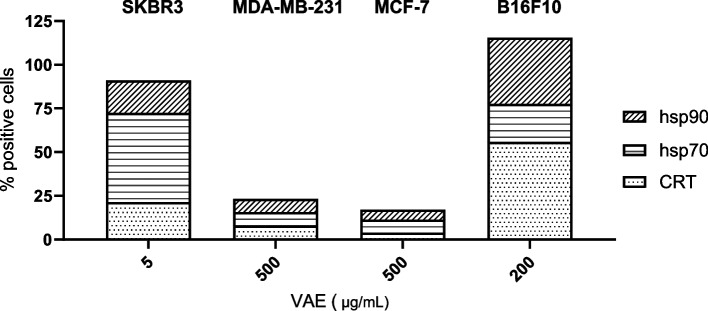
Proportion of viable cells displaying DAMP translocation to the cell surface following VAE treatment for 48 h. The results for each cell line are presented as the mean percentage from a total of at least three independent experiments

In SKBR3 cells, CRT and hsp70 were significantly increased after 24 h treatment with TM and taxol, and hsp90 was significantly increased by taxol. After 48 h treatment with TM and taxol or with 2.5 µg/mL VAE, all DAMPs were significantly increased (Fig. 4). The induction of a significant increase of DAMPs on the cell surface of MDA-MB-231 and MCF 7 cells by taxol and TM was different. VAE induced a significant increase in CRT, hsp70 and hsp90 in both MDA-MB-231 and MCF-7 cells after 48 h of treatment (Fig. 5). In MDA-MB-231 and MCF-7 cell lines, the number of cells with DAMPs translocated to the cell surface and the percentage of early apoptotic cells were considerably lower than in SKBR3 and B16F10, even after 48 h of VAE treatment. A concentration of 500 µg/mL VAE induced the translocation of DAMPs in 7–8% of treated 7-AAD/DAPI negative MDA-MB-231 cells (depending on the DAMP) and the percentage of early apoptotic cells was about 18%; in MCF-7, between 4 and 7% of cells were DAMP-positive and about 11% were early apoptotic. In contrast, 18–51% of viable SKBR3 cells treated with 5 µg/mL were DAMP-positive and 22% were early apoptotic. The B16F10 cell line had between 22 and 56% DAMP-positive cells and 52% early apoptotic cells after 48 h of VAE treatment at 200 µg/mL (Fig. 7). In addition, up to 50-fold higher concentrations of VAE were required to induce the appearance of DAMPs at the cell surface. This suggests that MCF-7 and MDA-MB-231 cells are more resistant to VAE. Nevertheless, we measured slightly but still significantly increased levels of DAMPs in the two cell lines after treatment with TM and VAE, and partially with taxol.

In B16F10 cells after 24 h, only 2.5 µM taxol significantly increased hsp70 and 100 µg/mL VAE significantly increased hsp90 levels (Fig. 6A), but 48 h treatment with taxol significantly increased the amount of CRT and hsp90 and treatment with TM significantly increased the exposure of hsp70 on the surface of early apoptotic cells. 100 µg/mL VAE significantly increased the exposure of all DAMPs at 48 h. (Fig. 6B). The 24 h and 48 h values for the more sensitive SKBR3 and B16F10 cells reflect the kinetics of cell surface DAMP exposure (Figs. 4 and 6).

**Fig. 8:**
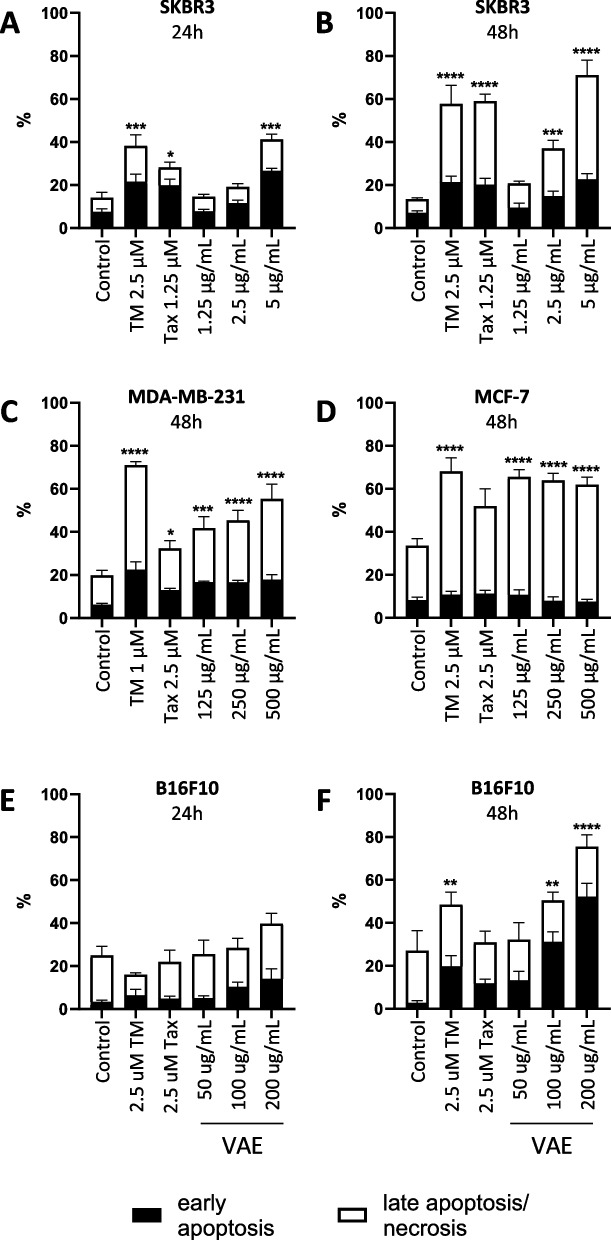
Cytotoxicity of **A** SKBR3 cells treated for 24 h, **B** SKBR3 cells treated for 48 h, **C** MDA-MB-231 cells treated for 48 h, **D** MCF-7 cells treated for 48 h, **E** B16F10 cells treated for 24 h and **F** B16F10 cells treated for 48 h with indicated concentrations of TM, taxol or VAE, respectively and untreated controls. Results are presented as mean percentage ± SE from at least 3 independent experiments. Significance values are given relative to untreated controls (*p < 0.05, **p < 0.01, ***p < 0.001, ****p < 0.0001). The indicated significance levels refer to the sum of early apoptotic and late apoptotic/necrotic cells

In SKBR3, B16F10 and MDA-MB-231, the amount of cell surface exposed CRT, hsp70 and hsp90, respectively, correlated significantly (*p* < 0.05 to *p* < 0.01, Spearman correlation) with the amount of early apoptosis at both 24 h and 48 h (see Supplementary Table 1 and Supplementary Fig. 3). Only the level of DAMPs on MCF-7 cell surface showed no significant correlation with early apoptosis. (Supplementary Table 1). This cell line showed a concentration-independent characteristically low level (< 10%) of early apoptosis and DAMP exposure, despite a high death rate after VAE, TM, or taxol treatment, respectively. Supplementary Fig. 4 shows representative dot blots of flow cytometric analysis of apoptosis and Fig. 8 shows apoptosis/viability data for all cell lines and treatments. Significance levels shown refer to the sum of all early apoptotic and late apoptotic/necrotic cells.

### Cellular HMGB1 and ATP release

Under our experimental conditions, no HMGB1 release was detected in the cell culture supernatants of SKBR3, MCF-7 and B16F10 cells (data not shown). Only in MDA-MB-231 cells treatment with the positive control mitoxantrone resulted in a significant increase in extracellular HMGB1 levels. It should be noted that this cell line was the only one that actively released HMGB1 into the supernatant even in the untreated control (see Supplementary Fig. 5).

**Fig. 9:**
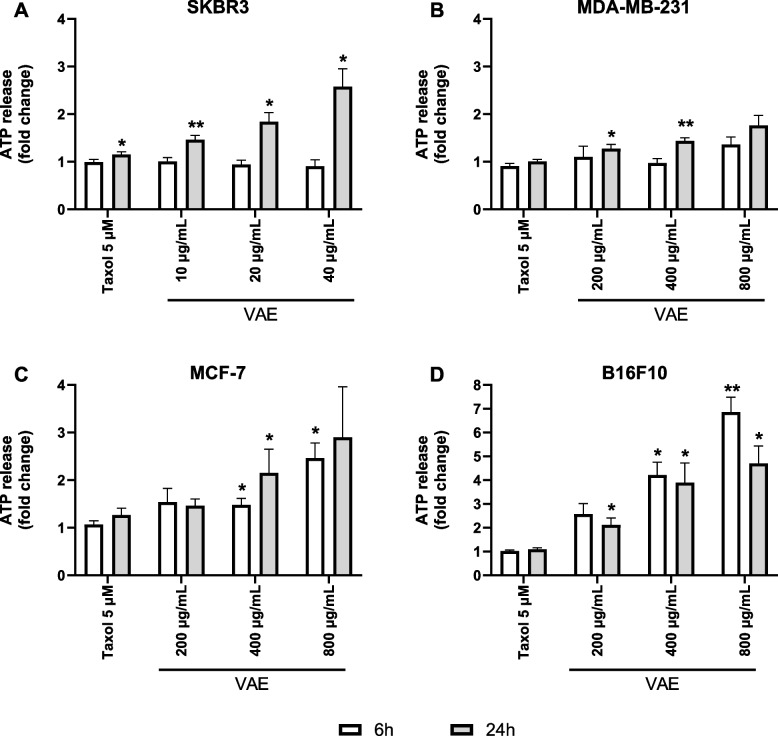
ATP release by **A** SKBR3, **B** MDA-MB-231, **C** MCF-7 and **D** B16F10 cells treated with indicated concentrations of taxol or VAE for 6 h and 24 h, respectively. Results are presented as the average change in extracellular ATP concentration compared to the untreated control (mean ± SE from at least 3 independent experiments). Significance values are expressed relative to untreated controls at 6 h and 24 h, respectively (*p < 0.05, **p < 0.01)

We measured the release of ATP in the cell culture supernatant after 6 and 24 h of treatment. As shown in Fig. 9, VAE induced a cell line, time- and concentration-dependent release of ATP from the tumor cells. In SKBR3 and MDA-MB-231 cells, no ATP release was observed after 6 h. In MCF-7 cells, ATP levels in the medium increased significantly compared to the untreated control after treatment with 400 and 800 µg/mL VAE for 6 h. In all three breast cancer cell lines, there was a significant up to threefold increase in ATP release after 24 h of VAE treatment. The effect of VAE treatment was most pronounced in the murine melanoma cell line B16F10. Extracellular ATP levels were significantly increased by up to sevenfold after 6 h of treatment with 400 and 800 µg/mL VAE and after 24 h of treatment with all VAE concentrations compared to the untreated control. A slightly increased significant ATP release after taxol treatment was observed only after 24 h of treatment of SKBR3 cells.

## Discussion

For an effective polyclonal adaptive immune response mediated by T and/or B cells against pre-existing tumor-specific or tumor-associated antigens, a sequence of events is essential, which Chen and Mellman have described as the cancer immunity cycle [11]. The first phase necessary to induce a specific antitumor immune response can be initiated by therapies that induce immunogenic cell death. Zhou et al. have reviewed several current and emerging therapeutic approaches to achieve this effect [32].

The aim of our project was to investigate whether treatment of tumor cell lines with an aqueous fermented mistletoe extract leads to cell death with the characteristics of immunogenic cell death. The positive controls were used for verification of the experimental procedures and will not be discussed further.

The induction of immunogenic cell death is mostly initiated by endoplasmic reticulum stress. Phosphorylation of eIF2α (eukaryotic initiation factor 2α) by the kinase PERK is a hallmark of ICD. Phosphorylation of eIF2α is a common stress response in eukaryotes. It leads to inactivation of global protein synthesis and subsequent signaling events such as activation of caspase-8, activation of the pro-apoptotic proteins BAX and BAK, and relocation of CRT to the cell surface [10]. Under our experimental conditions, we observed that treatment of cancer cell lines with VAE resulted in phosphorylation of EIF2α, but not in increased expression of ATF4 or CHOP. This result indicates an incomplete ER stress response, as previously shown by Bezu et al. for various chemotherapeutic and other agents [28]. Further specific stress inducers have been described, such as UV irradiation, which, despite robust phosphorylation of eIF2a, did not lead to an increase in ATF4 expression, but to its transcriptional repression. Tissue-specific regulation of ATF4 under certain stress conditions may also occur [33].

Little information is available on the induction of ER stress by mistletoe extracts. Park et al. showed that treatment of K562 cells with mistletoe extracts resulted in upregulation of GRP78/BiP expression, increased phosphorylation of eIF2α, reduction of p90 ATF6, and time-dependent induction of CHOP mRNA, indicating the induction of ER stress [34]. Maltseva et al. treated Molt4 and CaCo2 cells with mistletoe lectin I (MLI) and analyzed changes in the expression of genes involved in ER stress. Expression profiles were dependent on the cell type, MLI concentration and exposure time. They found increased levels of spliced XBP1 gene mRNA (XBP1 s), chaperone protein gene mRNA (HSPH1, HSPB1, and HSPA5), and transcription factor ATF6 gene mRNA, probably due to activation of IRE1a and ATF6-dependent signaling pathways. Changes in ATF4 and CHOP expression were dependent on MLI concentration [35].

We examined the production of superoxide in mitochondria by flow cytometry using the MitoSOX red assay. As a positive control, we used mito-paraquat (MitoPQ), a mitochondria-targeted agent that acts on mitochondrial complex I and leads to the production of superoxide [36]. ROS play a complex role in the regulation of pathways such as apoptosis, autophagy and necroptosis that are triggered by mitochondrial and ER stress [37, 38]. Both endogenous and exogenous stressors can induce ROS in a cell [39]. The main source of superoxide is leakage from the mitochondrial electron transport chain [40]. Damage to mitochondrial components by ROS can lead to loss of mitochondrial membrane potential, release of cytochrome c, further increase in cellular ROS, and apoptosis. The increased production of ROS is closely related to ER stress. Mitochondria-associated ER stress activates the specific danger signaling required to produce immunogenic DAMPs, and its induction is a powerful strategy for ICD enhancement and cancer immunotherapy [9, 41]. We measured the production of ROS to determine whether ROS may be involved in the VAE-induced processes leading to the expression of surrogate markers of ICD. We showed that VAE treatment significantly increased mitochondrial superoxide production in a concentration-dependent manner in all cell lines tested (Fig. 2). Korean mistletoe lectins induced the production of reactive oxygen species and the loss of mitochondrial membrane potential in hepatocarcinoma and macrophage-like cells [42, 43]. Mistletoe lectins (MLs) are mainly responsible for the cytotoxic and pro-apoptotic effects of VAE [44]. MLs belong to the ribosome-inactivating proteins class II (RIP-II), highly potent cytotoxins that interfere with protein biosynthesis and can induce different cell death mechanisms with different timing and relative potency in different cell lines. MLs are known to induce apoptosis via the intrinsic pathway. Mitochondrial damage leads to loss of mitochondrial membrane potential and activation of caspase-9. The downstream activated caspase-3 is an executor of DNA fragmentation, poly (ADP-ribose) polymerase (PARP) cleavage, and cell death by apoptosis. Bantel et al. showed that MLs can also activate caspase-8 in a death receptor-independent pathway, which may indicate an interplay between different death mechanisms [45, 46].

The major functions of the ER include calcium sequestration and glycoprotein processing. Chaperones are proteins within the ER that help fold newly synthesized proteins. They are involved in protein repair after cells are exposed to physical or chemical stress, during which they are rapidly increased. As a result of a stress response, chaperones may be translatorily upregulated, relocated to the cell surface, or released into the extracellular space. There, they promote the engulfment of dying cells ("eat-me"signals) and serve as a danger signal for the immune system, which can subsequently be activated [5, 47].

We measured the cell surface exposure of the DAMPs calreticulin and the heat shock proteins hsp70 and hsp90 after treatment with VAE or with tunicamycin or taxol. Calreticulin is one of the best-known DAMPs that is a major determinant in the process of immunogenic cell death. Calreticulin is expressed on the surface of most living cells and is involved in the removal of apoptotic cells (efferocytosis) by interacting with the internalization receptor LRP (LDL receptor-related protein, CD91) on the surface of phagocytes [48, 49]. Obeid et al. (2007) described the increased exposure of calreticulin on the cell surface of pre-apoptotic cells as a very early event of immunogenic cell death. Using mass spectrometry analysis, they demonstrated calreticulin exposure on the surface of CT26 mouse colon cancer cells treated with ICD inducers. This active translocation occurred before the first signs of apoptosis [50]. Panaretakis et al. showed that exocytosis of the calreticulin/ERp57 complex is accompanied by sequential activation of an ER stress response and activation of caspases prior to cell apoptosis [10].

In two of the four cell lines studied (SKBR3 and B16F10), we detected increased CRT exposure on the surface of pre-apoptotic cells (Fig. 3). This exposure of CRT prior to the appearance of phosphatidylserine is an important indication that VAE is capable of triggering ICD. As mentioned above, several factors are involved in the triggering of an ICD. Whether the biology of the individual cell lines, the experimental conditions, or other factors are responsible for the different results in the cell lines we studied must be clarified in future studies.

We observed a significant increase of the three DAMPs CRT, hsp70 and hsp90 on the surface of early apoptotic cells in 3 breast cancer and one murine melanoma cell line. For VAE, this effect was time-, concentration- and cell line-dependent. The Her-2-positive breast cancer cell line SKBR3 was the most sensitive, followed by the melanoma cell line B16F10. Significantly more resistant were the estrogen receptor positive cell line MCF-7 and the triple negative cell line MDA-MB-231, in which, despite statistical significance, there was only a small increase in the exposure of DAMPs on the early apoptotic cell surface.

Breast tumors are highly heterogeneous and can be classified based on several characteristics, including histological type, hormone receptor status, morphology and molecular profile. The cell lines we used can be classified into the following subtypes based on their molecular characteristics:

- SKBR3 (adenocarcinoma) – luminal Her2 +, claudin-low
- MCF-7 (invasive ductal carcinoma) – luminal A, estrogen receptor +, progesterone receptor +
- MDA-MB-231 (adenocarcinoma) – basal B, triple negative breast cancer, mesenchymal stem-like [51, 52]. 

VAE and particularly MLs do not appear to target specific tumor cell types, they bind to certain carbohydrate epitopes via their B-chain. Specifically, they bind to sialic acids, which are the part of glycoproteins present on the cell surface [53, 54]. During cancer progression and metastasis, the glycosylation of cancer cells changes. Sialic acids can be overexpressed compared to normal cells [55]. These changes in sialic acid expression may contribute to variations in the sensitivity of cell lines to VAE. It is known that MLs can influence the expression of genes involved in tumor cell survival, growth and metastasis. Treatment with mistletoe extracts has been shown to elicit specific gene expression profiles, such as immune or stress response genes, which in turn may preferentially trigger certain pathways [56]. Eggenschwiler et al. hypothesized that the higher the number of affected genes, the more sensitive a cell line is to the cytotoxic effect of VAE [57, 58]. Beztsinna et al. analyzed the cell binding, endocytosis, subcellular processing of MLs, and the activation of apoptosis [59]. It is important to note that these processes can vary depending on the specific cell line. Future studies must clarify resistance causes to mistletoe preparations.

The release of HMGB1 into the extracellular space by dying cells is considered one of the hallmarks of immunogenic cell death. HMGB1 can exert an immunostimulatory function via specific receptors such as RAGE and TLR4 [60, 61]. In our study, this molecular signal was not measurable by in vitro treatment of cancer cells with VAE. It is generally believed that HMGB1 is released only during necrotic cell death and not by apoptosis, because under physiological conditions, nuclear components like HMGB1 are retained during apoptosis until they are cleared by phagocytes [62]. Since the lectin-containing VAEs induce apoptosis, this may explain why HMGB1 was not detectable in our cell culture supernatants. Bell et al. though have shown that HMGB1 can also be released during late apoptosis [63]. If this was the case in our experiments, the HMGB1 levels were below the detection limit of the ELISA kit. The pro-inflammatory properties of mistletoe lectins themselves may at least partially compensate for the absence of HMGB1. Kim et al. showed that in vitro treatment of bone marrow-derived dendritic cells with the B chain of KML resulted in their maturation and the expression of co-stimulatory molecules and pro-inflammatory cytokines. These effects were found to be TLR4-dependent. The authors demonstrated the stimulatory effect of KML-B matured DCs on activation and proliferation of specific cytotoxic T cells and their INF-γ secretion. In vivo, they demonstrated the reduction of the tumor size in KML-B treated vaccinated mice [64, 65].

Another key feature of ICD is the release of ATP by dying cells which acts as both a "find me" signal and a potential inflammatory stimulus for DCs [66, 67]. After treatment with VAE, we measured significantly elevated ATP levels in the cell culture supernatants of all cell lines tested whereby the effect of VAE on the murine melanoma cell line was considerably stronger than on the breast cancer cell lines. Martins et al. showed that ATP is secreted via active exocytosis and in an autophagy- and caspase-dependent manner [66]. In the scientific literature we have not found any reports on the effect of mistletoe extracts on autophagy or on the release of ATP by treated cells. For other plant lectins, however, autophagy inducing effects have been demonstrated [68, 69]. The processes of autophagy and apoptosis are closely related [70]. Each can promote or inhibit the other. If the level of autophagy becomes too high, apoptosis will occur. This means that although the commonly known cell death induced by MLs is via the intrinsic apoptotic pathway, autophagy-dependent ATP release is possible at an early stage of VAE treatment. The precise impact of VAE on autophagy remains to be demonstrated in further studies.

The ICD-associated DAMPs promote the recruitment as well as the maturation and activation of DCs. Similar effects have been described in studies of the direct effect of European and Korean mistletoe extracts on DCs in vitro. Treatment of monocyte-derived immature DCs with VAE in vitro resulted in increased expression of HLA-DR, CD40, CD80, CD83, and CD86 and induction of the inflammatory cytokines IL-6, IL-8, IL-12, and TNF-a. In an allogeneic mixed lymphocyte reaction, specific CD8 T cells were activated and IFN-γ secretion was increased [71–73].

Furthermore, MLs are immunomodulators that induce the production of cytokines and other mediators. In breast cancer patients, serum IL-12 levels increased after mistletoe therapy, and peripheral blood mononuclear cells showed increased production of IFN-γ and IL-2 [74]. In another study, increased levels of IL-6 and IFN-γ were measured in the serum of mistletoe-treated breast cancer patients [75]. In B16 F10 implanted mice, VAE caused inhibition of tumor growth and upregulation of splenocyte proliferation and IL-12 secretion [76].

Our results, together with the above-mentioned pro-inflammatory properties of VAE led us to hypothesize that local mistletoe treatment could induce an ICD and lead to the stimulation of an antitumor immune response. This hypothesis is supported by the fact that intralesional mistletoe treatment has shown positive effects in clinical use. The (off-label) peri- or intratumoral treatment with mistletoe extracts has already been used in individual cases with promising results. Clinical case reports illustrate the effect of intratumoral mistletoe treatment on patients with cutaneous squamous cell carcinoma, adenoid cystic carcinoma, malignant pleural mesothelioma, diffuse large B-cell lymphoma, cervical carcinoma and carcinoma in situ, skull metastases, and colon carcinoma [77–83]. Intratumoral mistletoe treatment, sometimes in combination with systemic chemotherapy, has also been used in inoperable pancreatic cancer. No serious side effects were observed, and the treatment was considered feasible and safe [84]. Another clinical case report describes the complete remission of a recurrent duodenal carcinoma after intratumoral mistletoe therapy [85]. The effects and side effects of intraperitoneal, intrapleural, and intratumoral mistletoe therapy were studied and summarized in a multicenter observational study and prospective trials were recommended [86, 87]. To date, there have been no major studies on the use of intratumoral mistletoe therapy. Many studies including meta-analyses for the clinical use of mistletoe therapy can be found on the Mistletoe-therapy online database [88].

Our investigation showed that in vitro treatment of various breast cancer and melanoma cell lines elicited several pathways and features typical of immunogenic cell death.

However, it is important to consider further aspects contributing to a successful induction of an anti-tumor immune response. In this regard, the biology of individual tumors appears to be important and could have a significant impact on a successful ICD induction.

## Conclusions

Our study showed that in vitro treatment of various breast cancer and melanoma cell lines induced several stress-related molecular processes characteristic of immunogenic cell death: The induction of p-eIF2α indicated the triggering of an ER stress response and we proved increased mitochondrial ROS production, calreticulin exposure on the surface of pre-apoptotic cells and cell surface expression or release of the DAMPs calreticulin, hsp70, hsp90 and ATP.

Our results encourage further studies to evaluate the effect of mistletoe-induced cancer cell death on the activation of an adaptive anti-tumor immune response.

## Supplementary Information

Supplementary Material 1.

Supplementary Material 2.

Supplementary Material 3.

## Data Availability

All data generated or analyzed during this study are included in this published article and its supplementary information files. The datasets generated for this study are available on reasonable request from the corresponding author.

## Abbreviations

ICD: Immunogenic cell death
DAMPs: Damage-associated molecular patterns
eIF2α/p-eIF2 α: (Phospho)-eukaryotic translation initiating factor 2A
ATF4: Activating transcription factor 4
CHOP: C/EBP-homologous protein
Hsp: Heat shock protein
ROS: Reactive oxygen species
CRT: Calreticulin
VAE: Viscum album extract
ER: Endoplasmic reticulum
HMGB1: High-mobility group protein B1
ATP: Adenosine triphosphate
IRE1α: Inositol-requiring enzyme 1α
PERK: Protein kinase R-like endoplasmic reticulum kinase
ATF6: Activating transcription factor 6
UPR: Unfolded protein response
DCs: Dendritic cells
ML: Mistletoe lectin
MSR: MitoSOX red
BAX: Bcl-2–associated X protein
BAK: Bcl-2 homologous antagonist
XBP1: X-box binding protein 1
RIP-II: Ribosome-inactivating protein class II
PARP: Poly (ADP-ribose) polymerase
LRP: LDL receptor-related protein
RAGE: Receptor for advanced glycation endproducts
TLR4: Toll-like receptor 4
KML: Korean mistletoe lectin
