# Supplementary material for: A fermented Mistletoe (Viscum album L.) extract elicits markers characteristic for immunogenic cell death driven by endoplasmic reticulum stress in vitro
Source: BMC Complement Med Ther. 2025 May 14;25:175. doi: 10.1186/s12906-025-04909-8 (PMC12076857; doi:10.1186/s12906-025-04909-8)
Supplement: Supplementary file 1 — Supplementary Material 1. [file 12906_2025_4909_MOESM1_ESM.pdf]

A Fermented Mistletoe (*Viscum album* L.) Extract Elicits markers characteristic for Immunogenic Cell Death Driven by Endoplasmic Reticulum Stress *in vitro*

Ulrike Weissenstein[1*], Sibylle Tschumi[1], Bettina Leonhard[1], Stephan Baumgartner[1,2]

[1] Society for Cancer Research, Arlesheim, Switzerland

[2] Institute of Integrative Medicine, Witten/Herdecke University, Herdecke, Germany

* Corresponding Author

Supplementary Material

Gating strategy for DAMP analysis

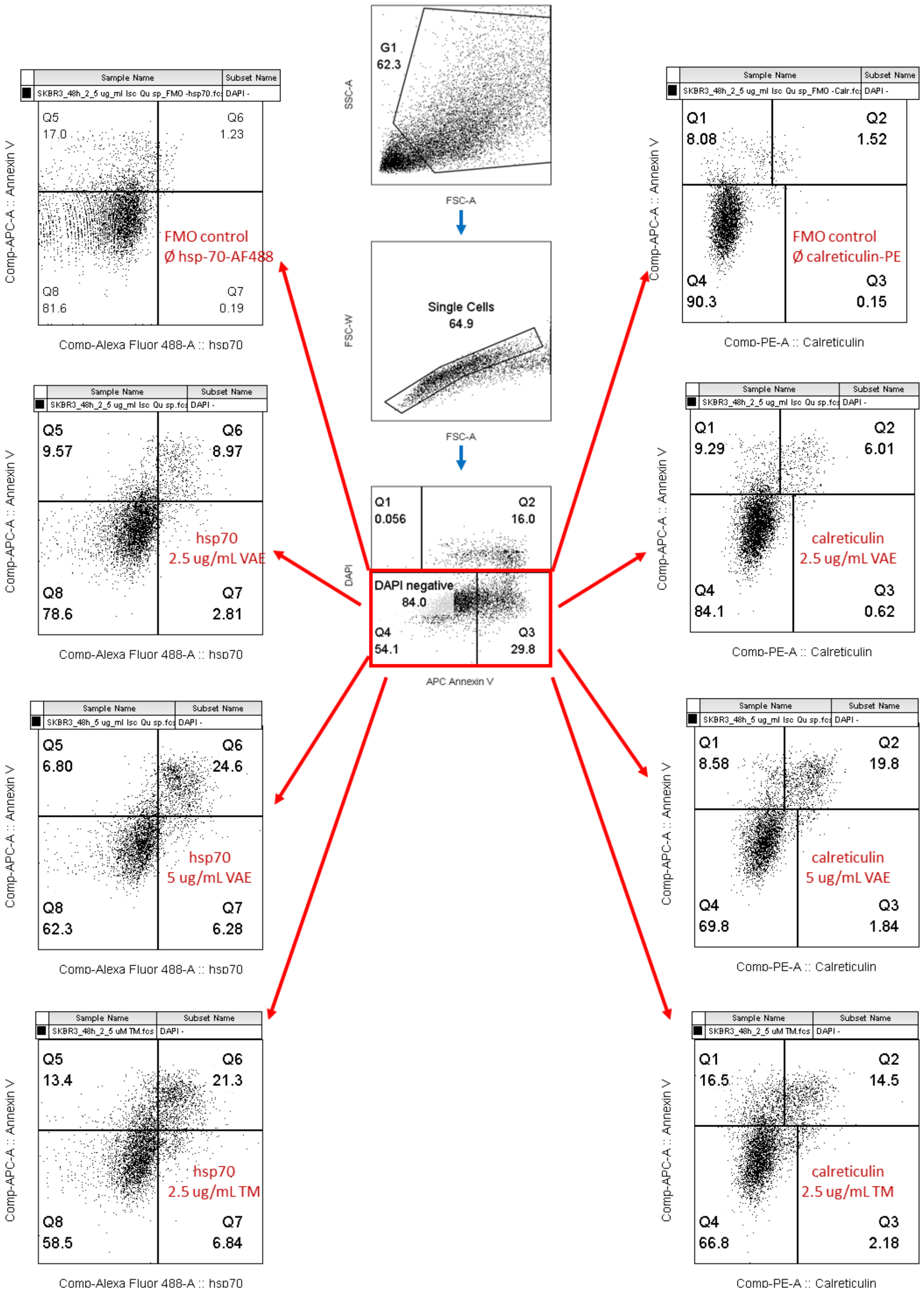

Supplementary Figure 1. Representative flow cytometry plots illustrating the gating strategy for FACS analysis of cancer cells. In the FSC/SSC dot blot, G1 encompasses the cancer cell population, single cells were further gated. Dapi negative cells were then analyzed for their expression of Annexin V and the respective DAMPs. Separation between DAMP-positive and DAMP-negative populations was performed using FMO (fluorescence minus one) controls.

Flow cytometry analysis of DAMP exposure

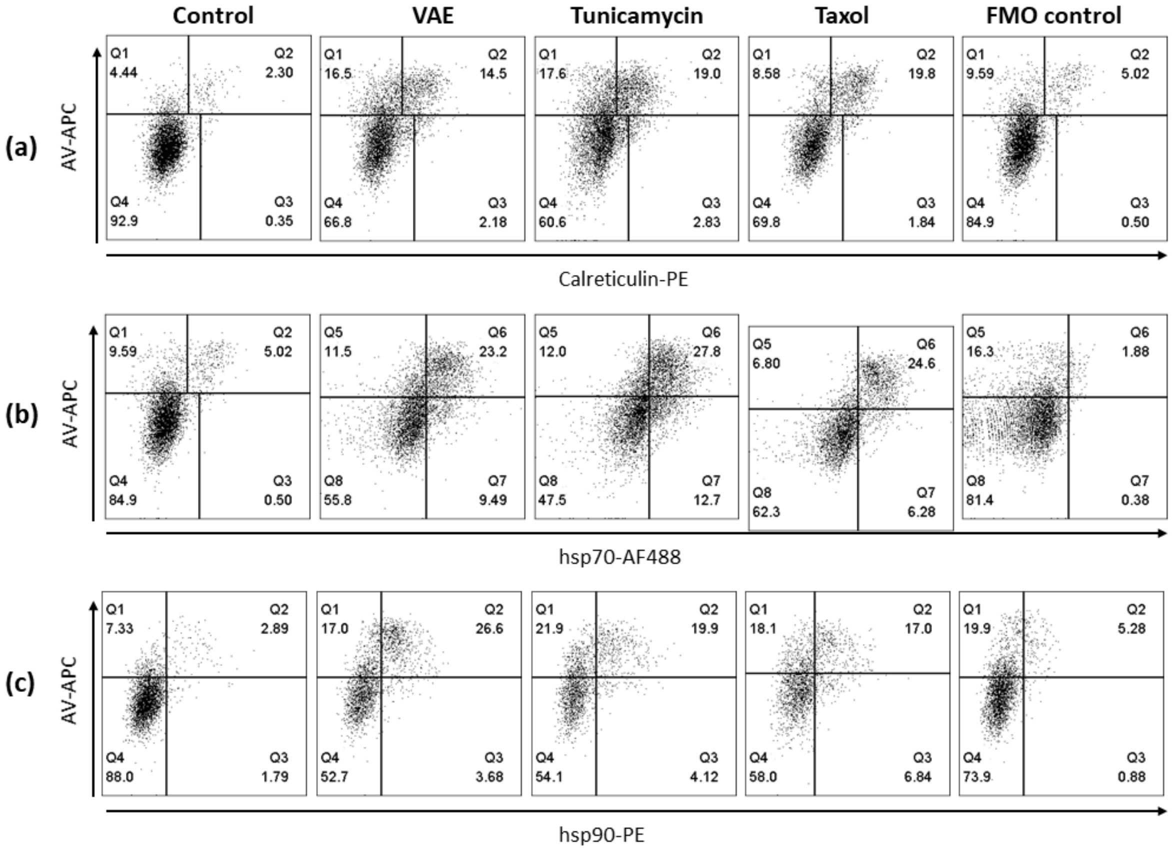

Supplementary Figure 2. Representative dot blots of the flow cytometry analysis of DAMP exposure on the surface of 7-AAD/DAPI-negative SKBR3 cells after 48h treatment with VAE, tunicamycin or Taxol compared to untreated control and FMO control. (a) Calreticulin, (b) hsp70 and (c) hsp90.

Spearman nonparametric correlation between early apoptosis and surface expositions of DAMPs

Supplementary Table 1: Spearman nonparametric correlation between early apoptosis and surface expositions of DAMPs after 24h treatment (SKBR3 and B16F10) and 48h treatment (SKBR3, MDA-MB-231, MCF-7 and B16F10) with ICD inducing drugs

		**AV+/DAPI- vs. CAL+**	**AV+/DAPI- vs. hsp70+**	**AV+/DAPI- vs. hsp90+**
**SKBR3 24h**	Spearman r	0.9429	0.8857	1.0
	P value (two-tailed)	0.0167	0.0333	0.0028
	Exact or approximate P value	Exact	Exact	Exact
	Significance level (alpha = 0.05)	Yes	Yes	Yes
**SKBR3 48h**	Number of XY Pairs	6	6	6
	Spearman r	1.000	0.9429	0.9429
	P value (two-tailed)	0.0028	0.0167	0.0167
	Exact or approximate P value	Exact	Exact	Exact
	Significance level (alpha = 0.05)	Yes	Yes	Yes
	Number of XY Pairs	6	6	6
**MDA-MB-231 48h**	Spearman r	1.000	0.9429	0.9429
	P value (two-tailed)	0.0028	0.0167	0.0167
	Exact or approximate P value	Exact	Exact	Exact
	Significance level (alpha = 0.05)	Yes	Yes	Yes
	Number of XY Pairs	6	6	6
**MCF-7 48h**	Spearman r	-0.2000	-0.2000	0.2571
	P value (two-tailed)	0.7139	0.7139	0.6583
	Exact or approximate P value	Exact	Exact	Exact
	Significance level (alpha = 0.05)	No	No	No
	Number of XY Pairs	6	6	6

**B16F10 24h**	Spearman r	0.9429	0.8857	0.9429
	P value (two-tailed)	0.0167	0.0333	0.0167
	Exact or approximate P value	Exact	Exact	Exact
	Significance level (alpha = 0.05)	Yes	Yes	Yes
	Number of XY Pairs	6	6	6
**B16F10 48h**	Spearman r	1.000	0.8286	0.9429
	P value (two-tailed)	0.0028	0.0583	0.0167
	Exact or approximate P value	Exact	Exact	Exact
	Significance level (alpha = 0.05)	Yes	No	Yes
	Number of XY Pairs	6	6	6

Correlation of Apoptosis and DAMP surface expression

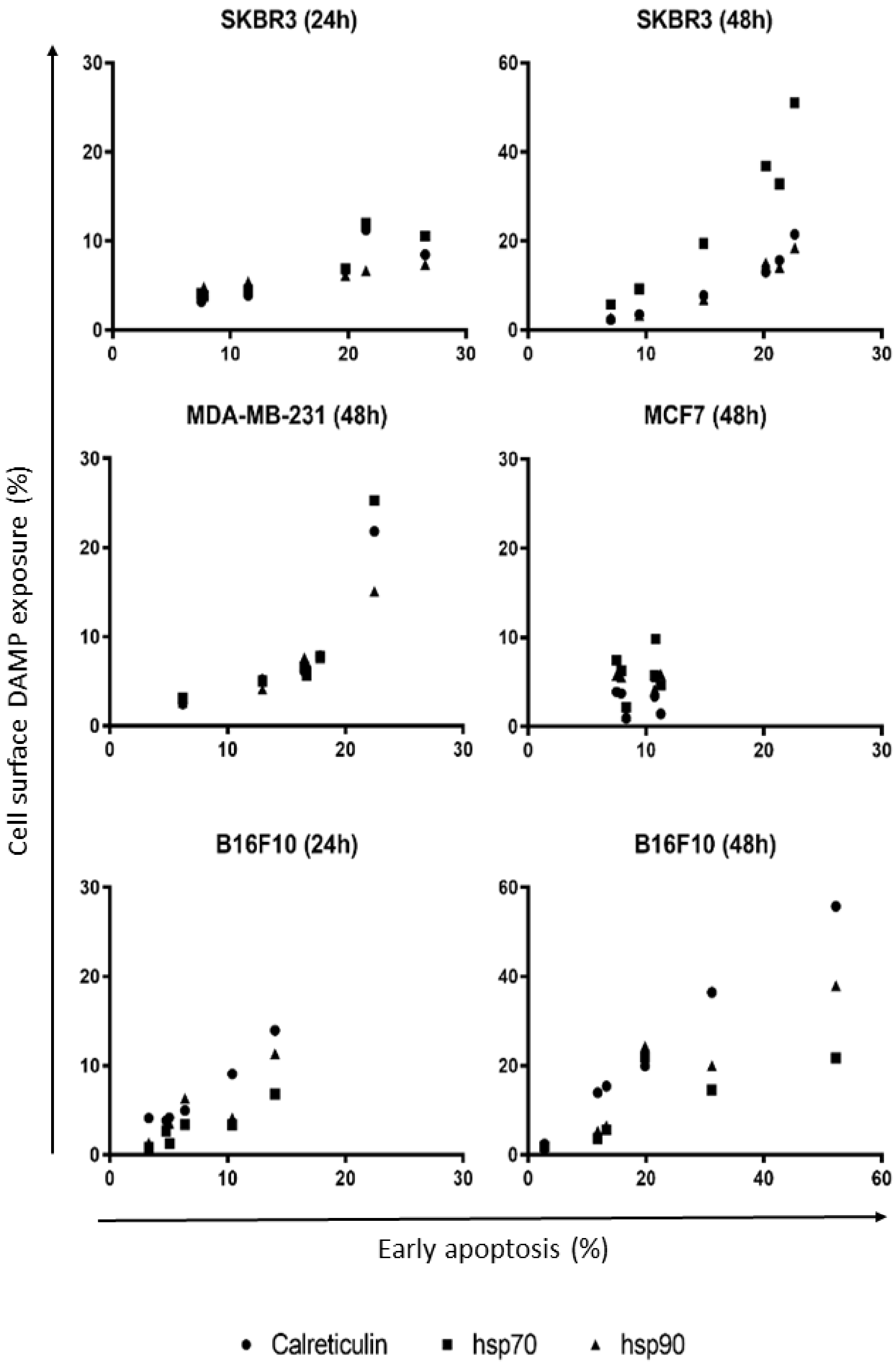

Supplementary Figure 3. Scatter plot of representative correlation between the cell surface DAMP exposure and early apoptosis detected in cancer cell lines

Flow cytometric analysis of apoptosis

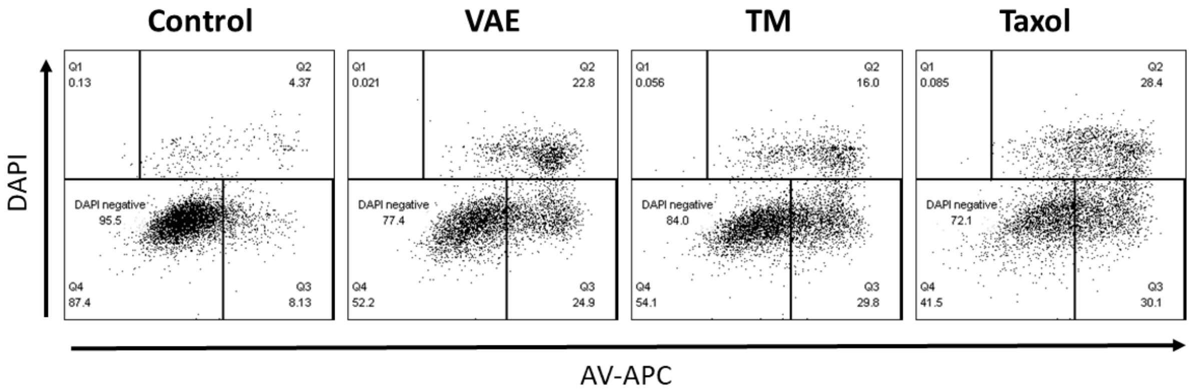

Supplementary Figure 4. Representative dot blots of flow cytometric analysis of apoptosis in SKBR3 cells treated for 48h with VAE, tunicamycin or Taxol compared to untreated control. Cells were labeled with Annexin V-APC and DAPI. The numbers in the graphs represent the percentages of cells in each quadrant. Q1 and Q2: late apoptotic/necrotic cells, Q3: early apoptotic cells, Q4: living cells. Cells in Q3 and Q4 were referred to as DAPI negative.

HMGB1 release by MDA-MB-231 cells

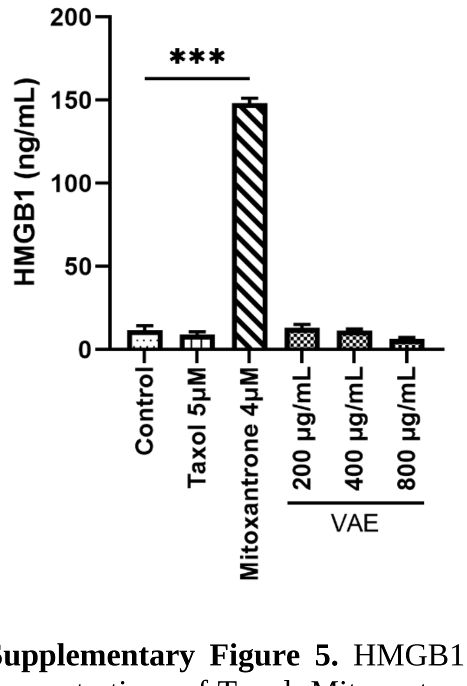

Supplementary Figure 5. HMGB1 release by MDA-MB-231 cells. Cells treated with indicated concentrations of Taxol, Mitoxantrone or VAE for 24h. Results are presented as mean ± SE from at least 3 independent experiments. Significance values are given relative to the untreated control (***p<0.001).
