# Supplementary material for: A fermented Mistletoe (Viscum album L.) extract elicits markers characteristic for immunogenic cell death driven by endoplasmic reticulum stress in vitro
Source: BMC Complement Med Ther. 2025 May 14;25:175. doi: 10.1186/s12906-025-04909-8 (PMC12076857; doi:10.1186/s12906-025-04909-8)
Supplement: Supplementary file 2 — Supplementary Material 2. [file 12906_2025_4909_MOESM2_ESM.pdf]

A Fermented Mistletoe (*Viscum album* L.) Extract Elicits markers characteristic for Immunogenic Cell Death Driven by Endoplasmic Reticulum Stress *in vitro*

Ulrike Weissenstein[1*], Sibylle Tschumi[1], Bettina Leonhard[1], Stephan Baumgartner[1,2]

[1] Society for Cancer Research, Arlesheim, Switzerland

[2] Institute of Integrative Medicine, Witten/Herdecke University, Herdecke, Germany

* Corresponding Author: u.weissenstein@vfk.ch

Supplementary material_2: Western blot images

**Cell line**	**Exp.-No.**	**EIF2 α**	**Total protein**
SKBR3	**210505**<br>Lane A-D<br>24h<br>Lane E-H<br>48h	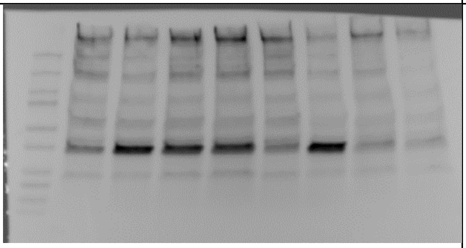	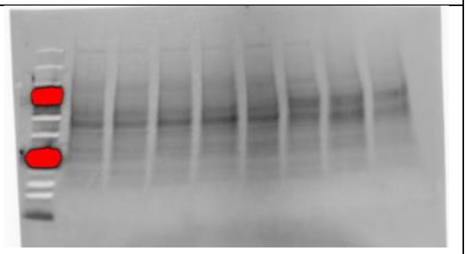
SKBR3	**210513**<br>Lane A-D<br>16h<br>Lane E-H<br>24h	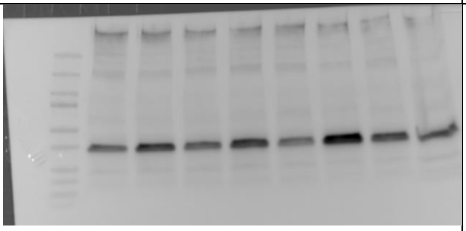	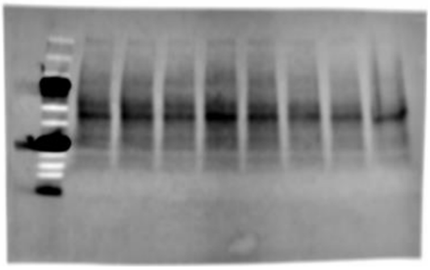
SKBR3	**210518**<br>Lane A-D<br>16h<br>Lane E-H<br>24h	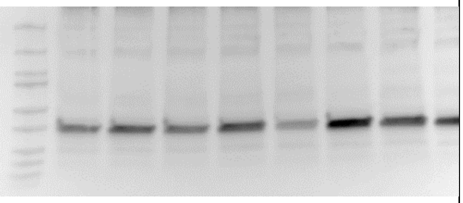	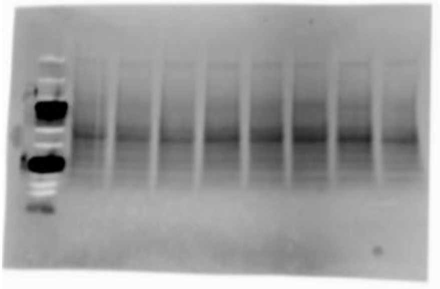

SKBR3	**220601**<br><br>(24h, more concentra-tions)	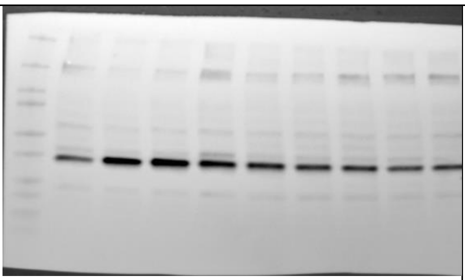	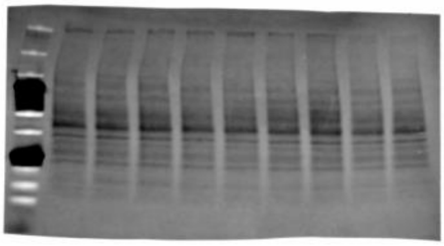
MCF-7<br>+ MDA-MB-231	**210615**	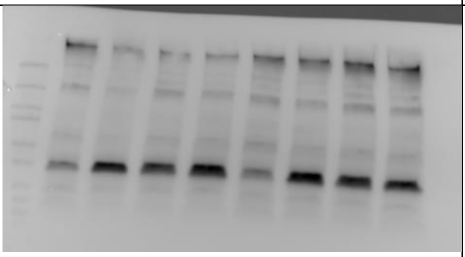	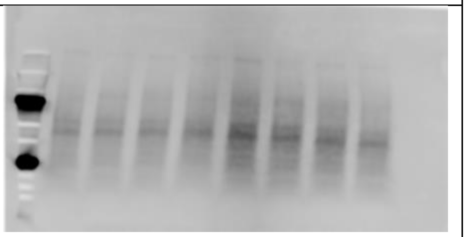
MCF-7<br>+ MDA-MB-231	**210713**	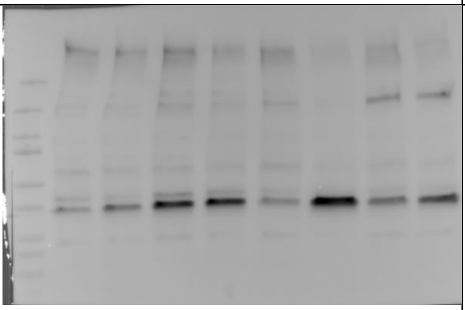	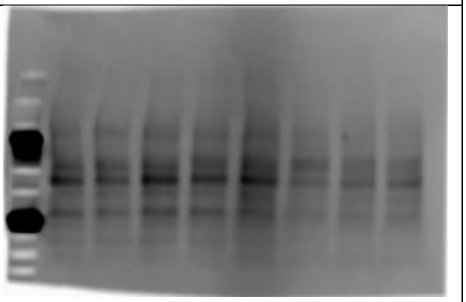
MCF-7<br>+ MDA-MB-231	**210719**	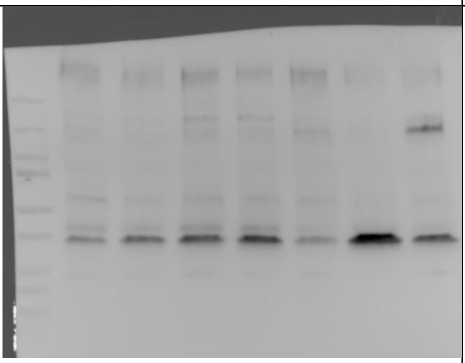	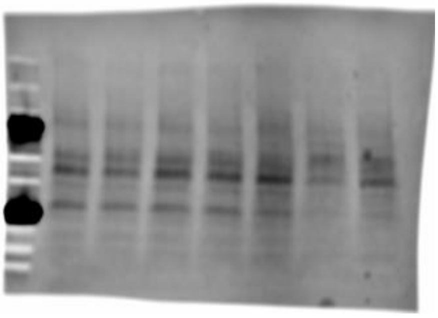
B16F10	**231115**	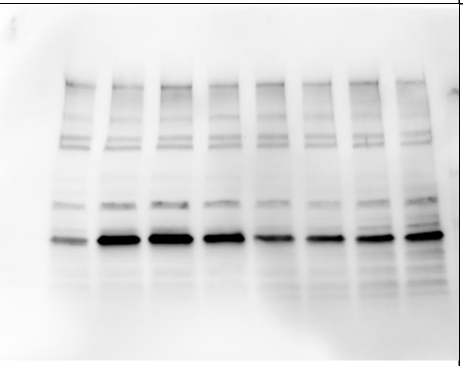	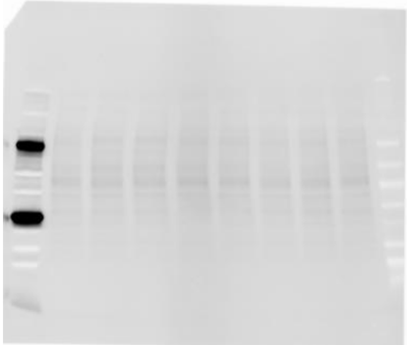

B16F10	**231129_WH**<br>**231205_WH**	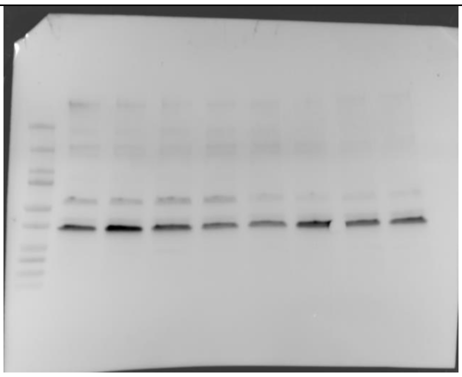	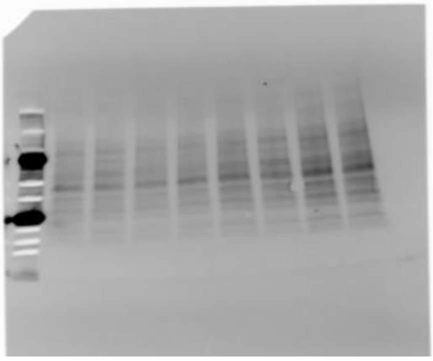
B16F10	**231213**<br>(Lane E-H)	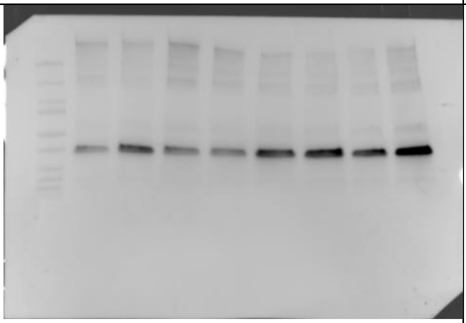	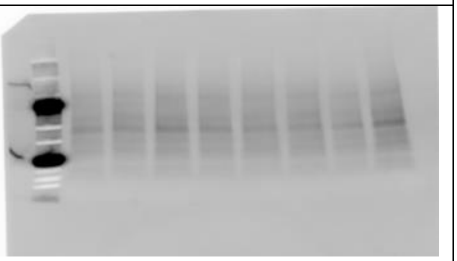
B16F10	**240110**<br>**240117**	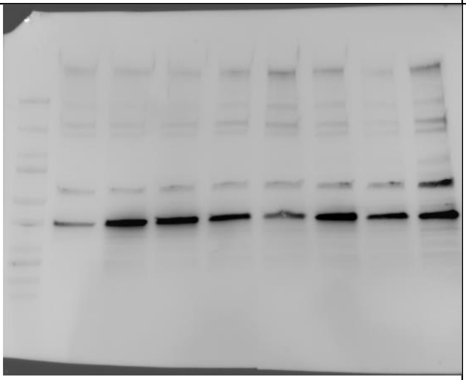	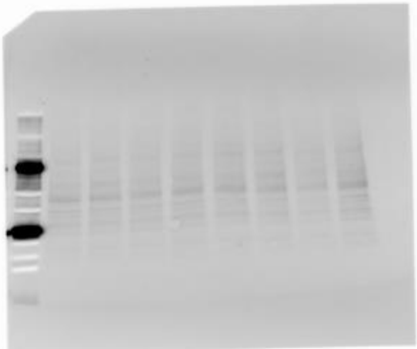

Cell line	Exp.-No.	ATF4	Total protein
SKBR3	240425<br>220601	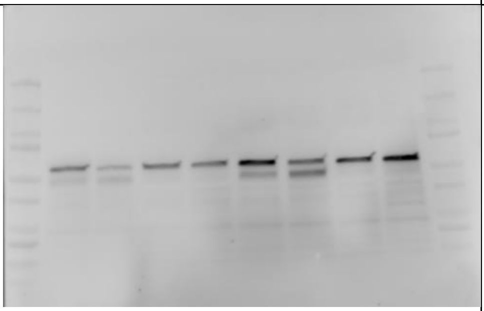	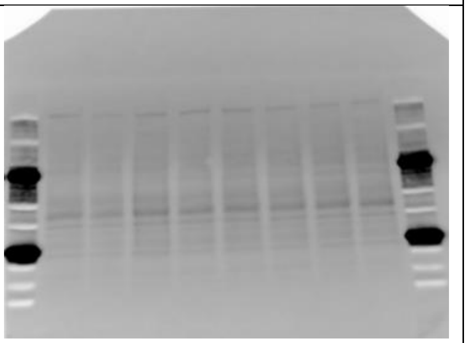
SKBR3	240228<br>240305	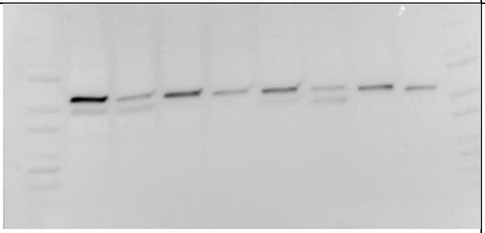	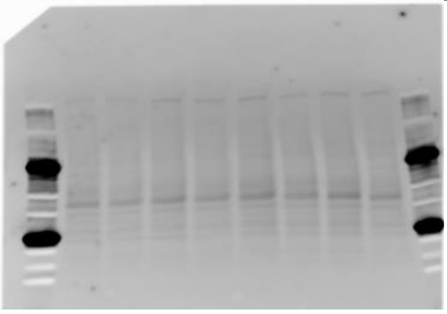
SKBR3	240424_1<br>240424_2	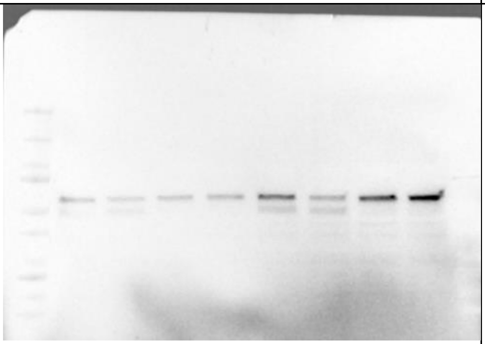	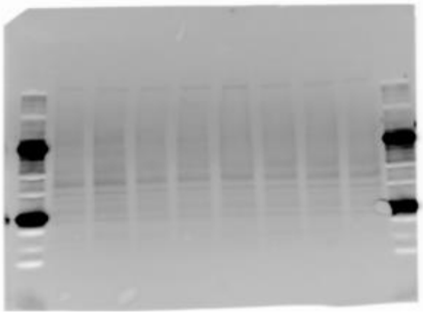
MCF-7 + MDA-MB-231	210615	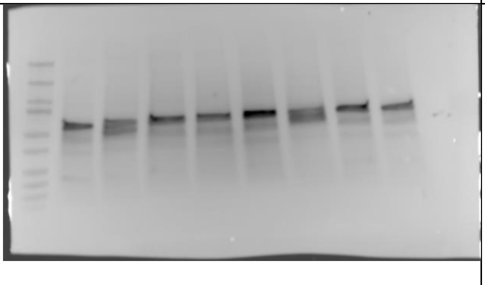	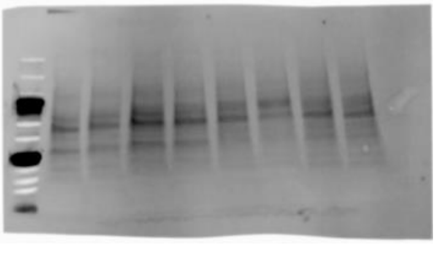
MCF-7 + MDA-MB-231	210713	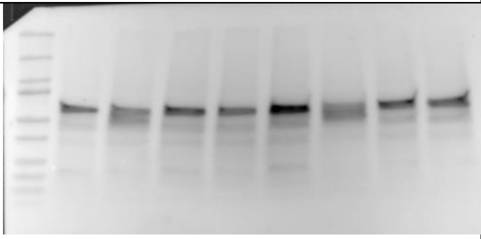	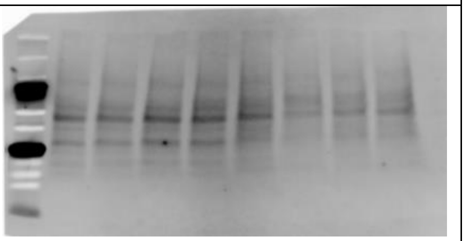

MCF-7 + MDA-MB-231	**210719**	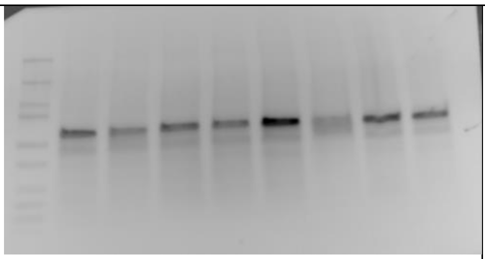	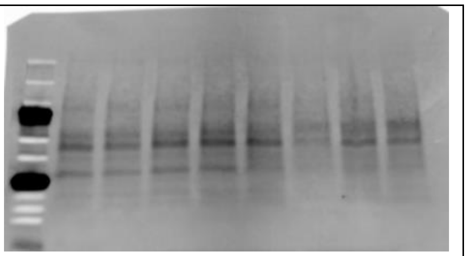
MCF-7	**210719**<br>**(repeat)**<br>**(2x)**	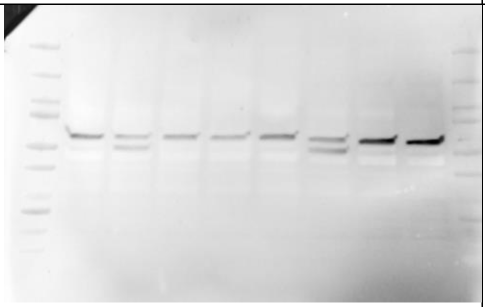	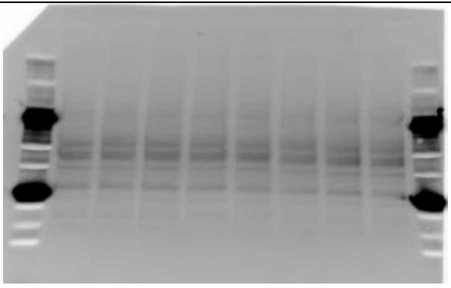
B16F10	**231115**<br>**231129**	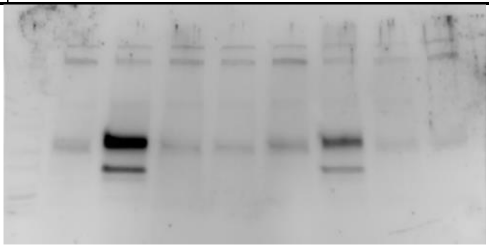	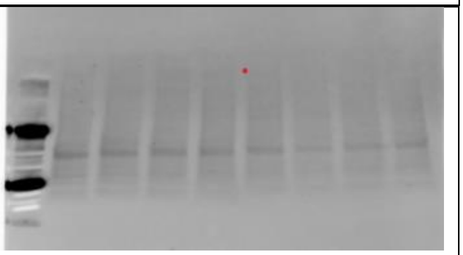
B16F10	**231205**<br>**231213**	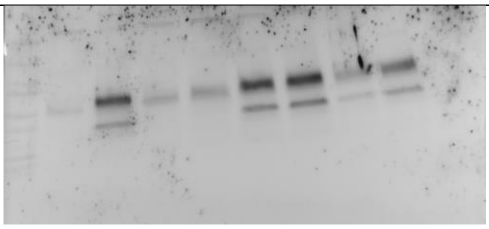	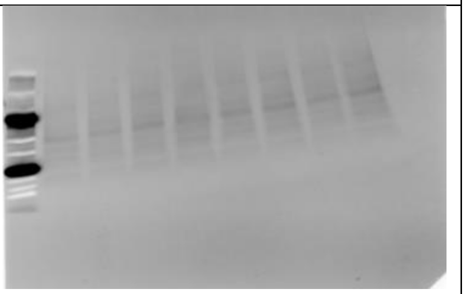
B16F10	**240110**<br>**240117**	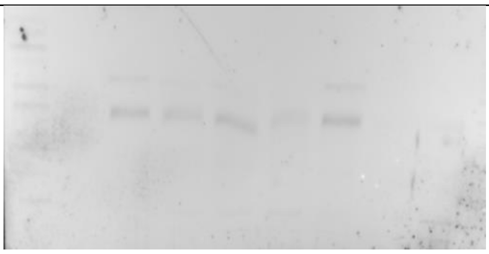	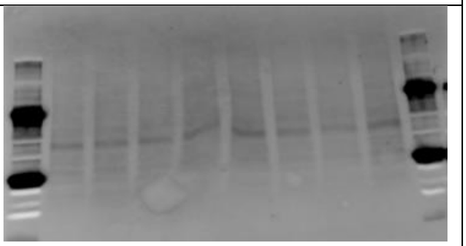

**Cell line**	**Exp.-No.**	**CHOP**	**Total protein**
SKBR3	**210505**<br>**210513**	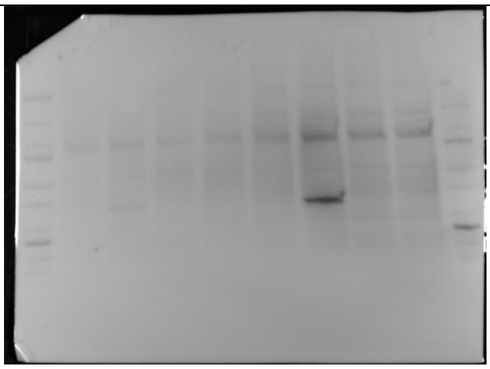	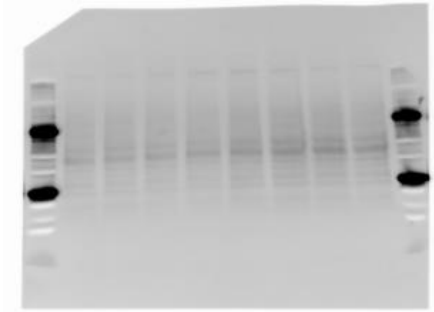
SKBR3	**210518**	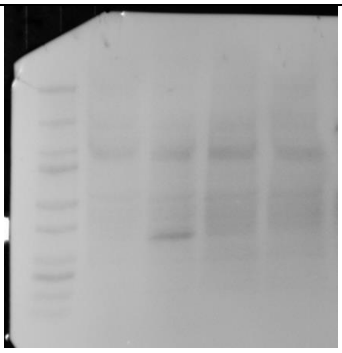	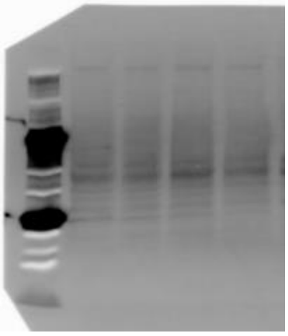
MDA-MB-231	**210615**<br>**210719**	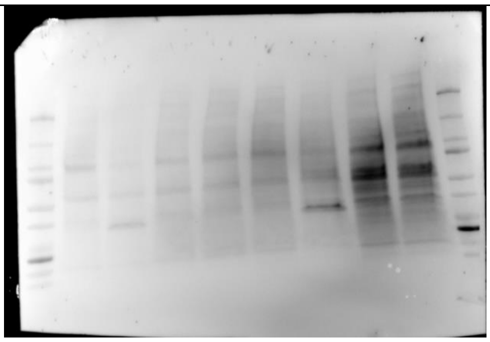	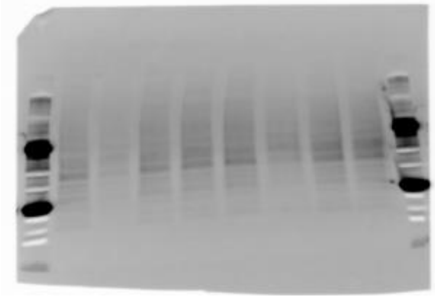
MDA-MB-231	**210713**<br>(Lane E-H)	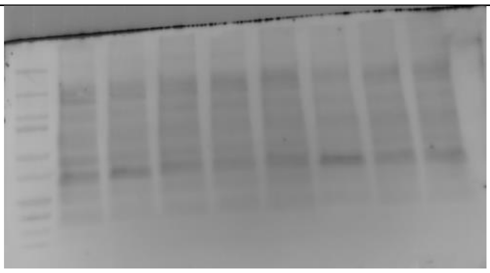	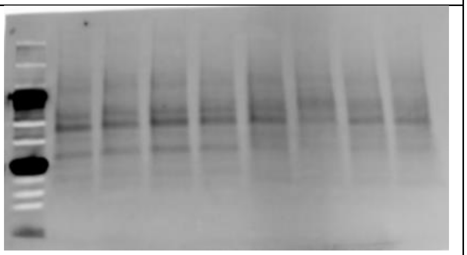

MCF-7	**2107615**	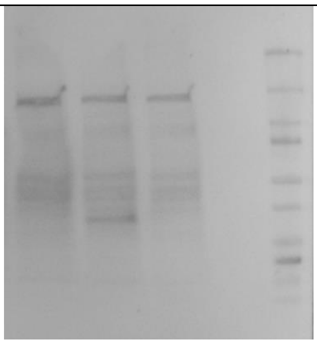	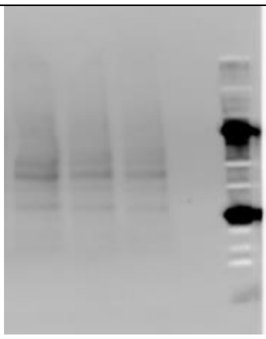
MCF-7	**210713**<br>**210719**	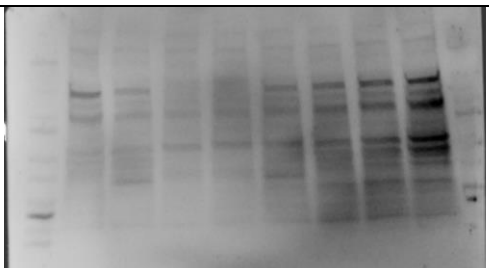	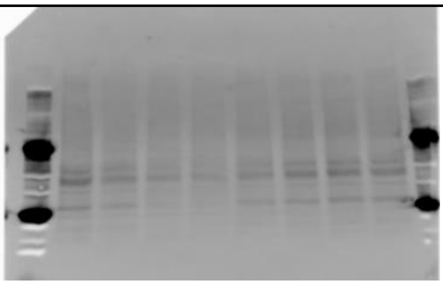
B16F10	**231115**<br>**231129**	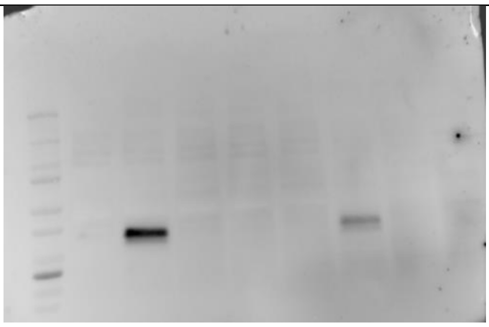	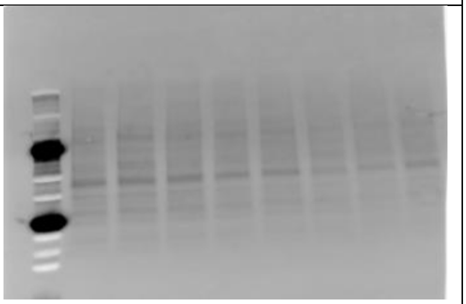
B16F10	**231205**<br>**231213**	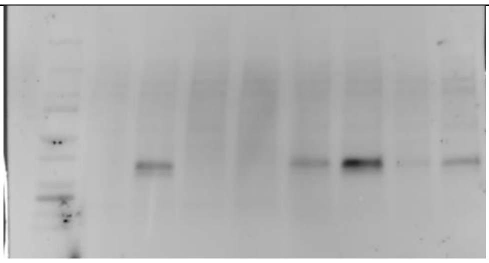	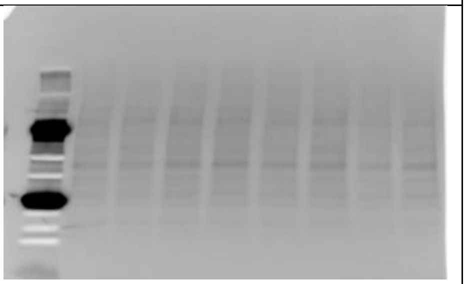
B16F10	**240110**<br>**240117**	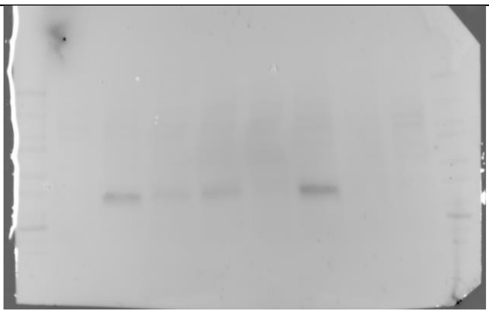	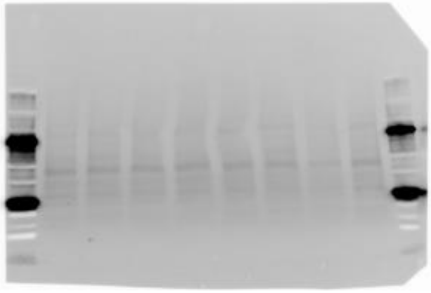
