# Supplementary material for: A fermented Mistletoe (Viscum album L.) extract elicits markers characteristic for immunogenic cell death driven by endoplasmic reticulum stress in vitro
Source: BMC Complement Med Ther. 2025 May 14;25:175. doi: 10.1186/s12906-025-04909-8 (PMC12076857; doi:10.1186/s12906-025-04909-8)
Supplement: Supplementary file 3 — Supplementary Material 3. [file 12906_2025_4909_MOESM3_ESM.pdf]

A Fermented Mistletoe (*Viscum album* L.) Extract Elicits markers characteristic for Immunogenic Cell Death Driven by Endoplasmic Reticulum Stress *in vitro*

Ulrike Weissenstein[1*], Sibylle Tschumi[1], Bettina Leonhard[1], Stephan Baumgartner[1,2]

[1] Society for Cancer Research, Arlesheim, Switzerland

[2] Institute of Integrative Medicine, Witten/Herdecke University, Herdecke, Germany

* Corresponding Author: u.weissenstein@vfk.ch

Supplementary material_3: Dose response data

The following pages provide figures with preliminary viability and dose-response data for the compounds used in the publication. These data were used to select the concentrations of the substances. The final VAE doses were selected based on effective concentrations in terms of DAMP expression. In addition, the dose-dependency had to be apparent. Calreticulin was primarily considered, but hsp70 and hsp90 were also assessed. Viability was considered to ensure that sufficient live cells were available for analysis. The concentration ranges for the control substances tunicamycin (TM), taxol (Tax) and MitoPQ were based on publications on the subject. In each case, 1-3 doses were tested and those that induced DAMP exposure were selected. Noticeably, the effects were variable with respect to different DAMPs and different cell lines. The statistical analyses shown in supplementary material_3 do not correspond to the final analyses, as some normalizations have been carried out there.

The order of figures is not the same as in the publication. The concentration ranges of the previous experiments were used for the ATP measurements.

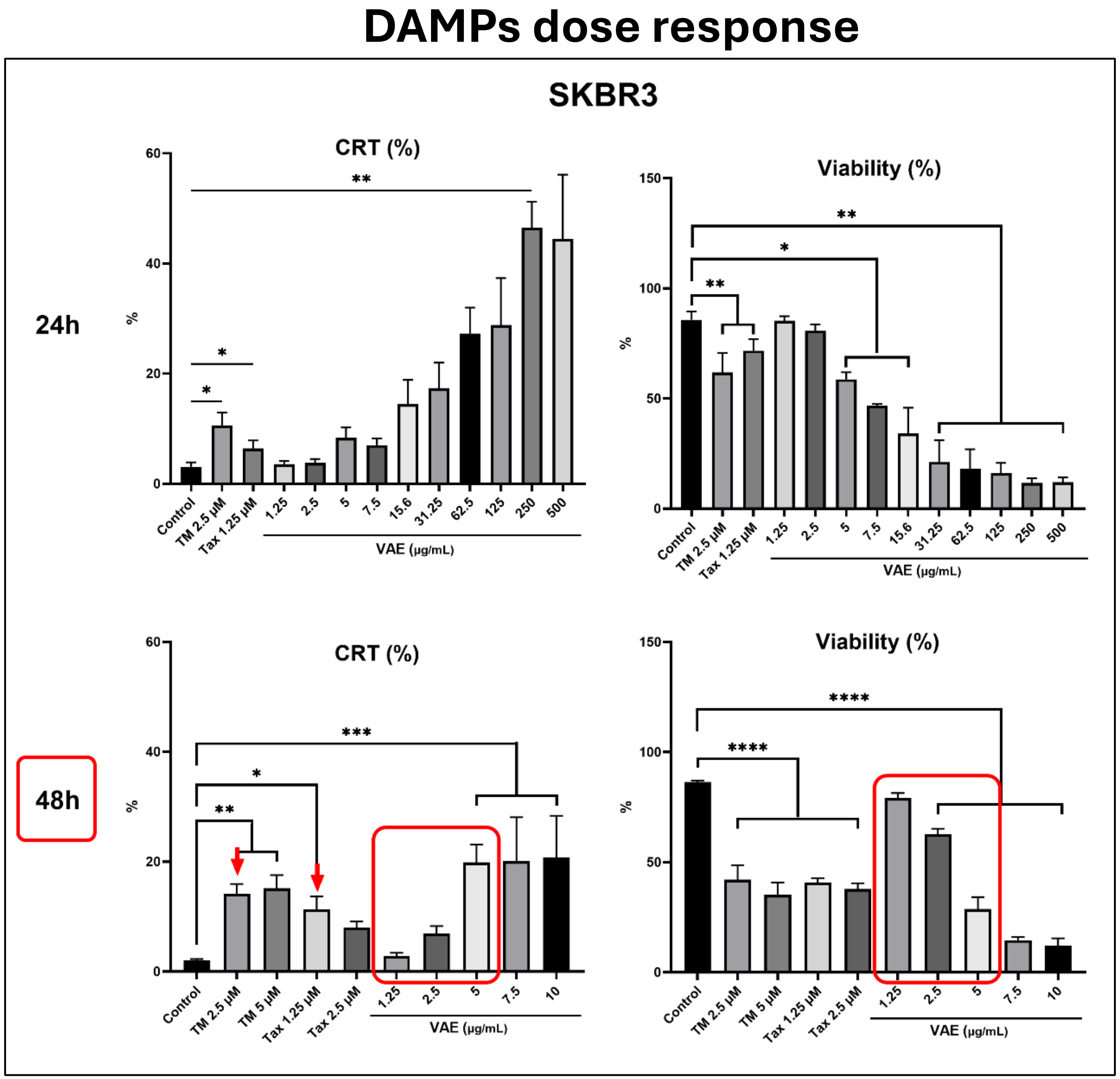

Suppl._3 Figure 1: Cell surface exposure of calreticulin (CRT) and viability after treatment of SKBR3 cells for 24h or 48h with either tunicamycin (TM), taxol (Tax) or viscum album extract (VAE). The areas outlined in red, or marked with red arrows, indicate the conditions that were used for the final analysis.

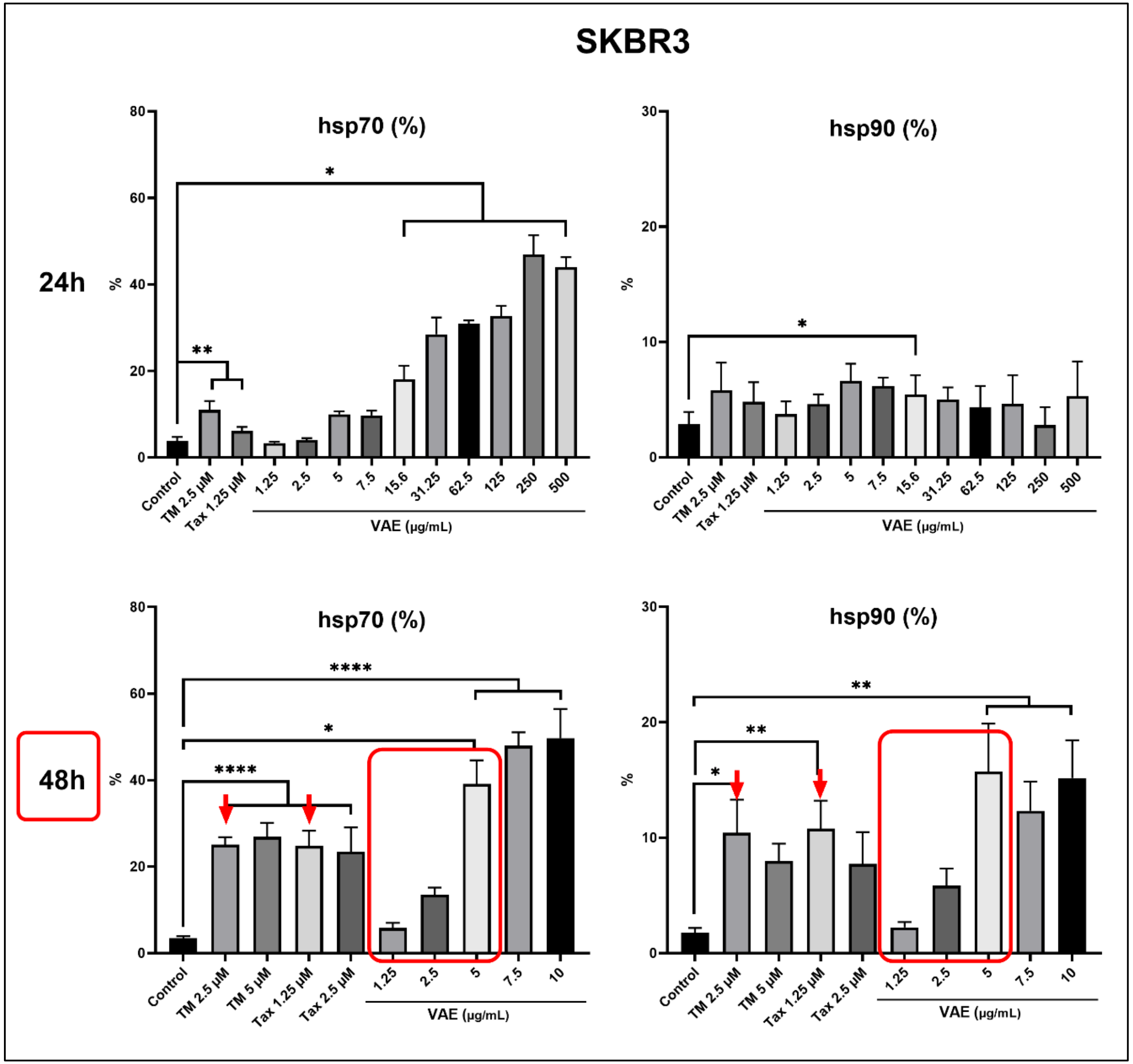

Suppl._3 Figure 2: Cell surface exposure of hsp70 and hap90 after treatment of SKBR3 cells for 24h or 48h with either tunicamycin (TM), taxol (Tax) or viscum album extract (VAE). The areas outlined in red, or marked with red arrows, indicate the conditions that were used for the final analysis.

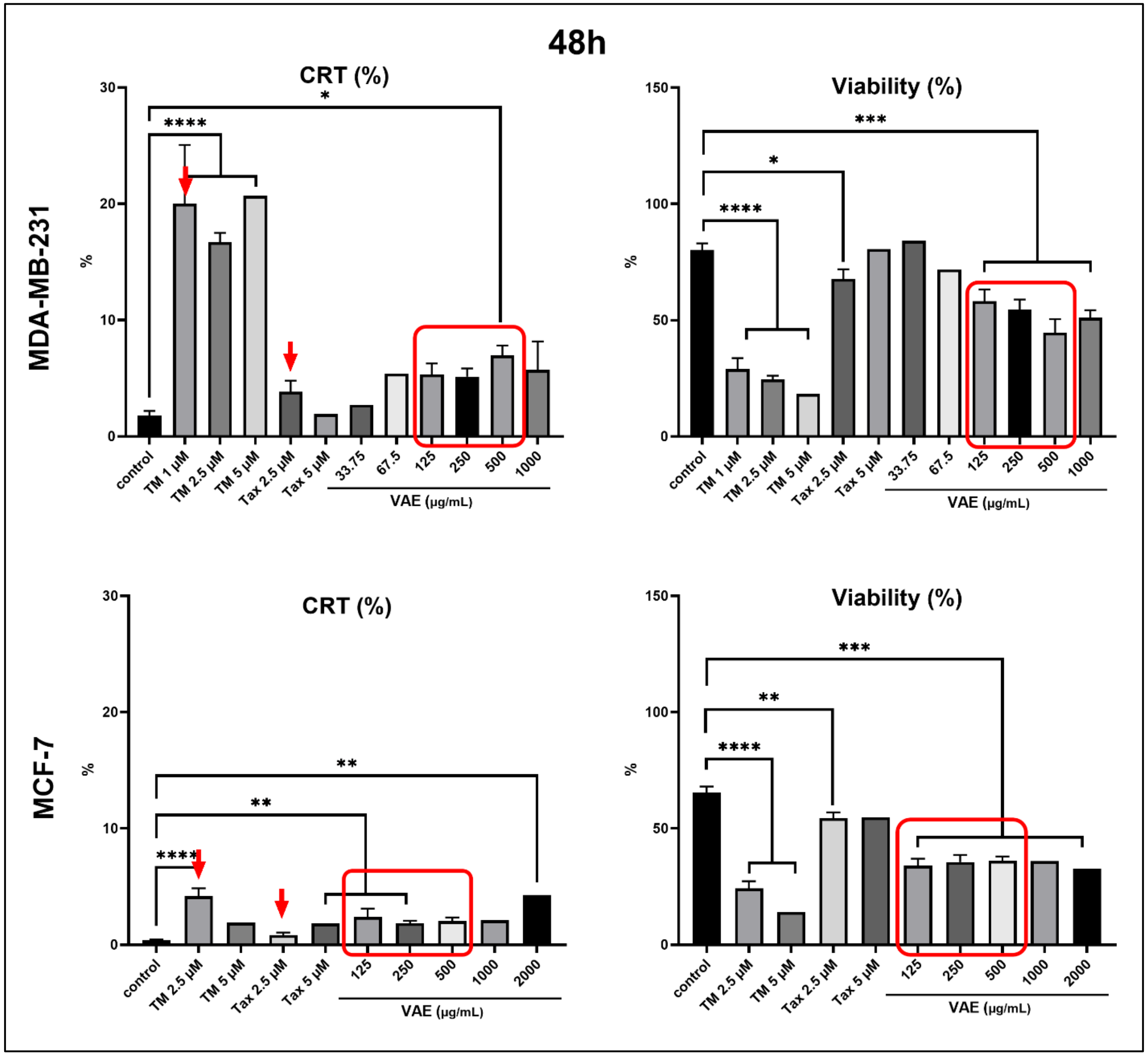

Suppl._3 Figure 3: Cell surface exposure of calreticulin (CRT) and viability after treatment of MDA-MB-231 and MCF-7 cells, respectively for 48h with either tunicamycin (TM), taxol (Tax) or viscum album extract (VAE). The areas outlined in red, or marked with red arrows, indicate the conditions that were used for the final analysis.

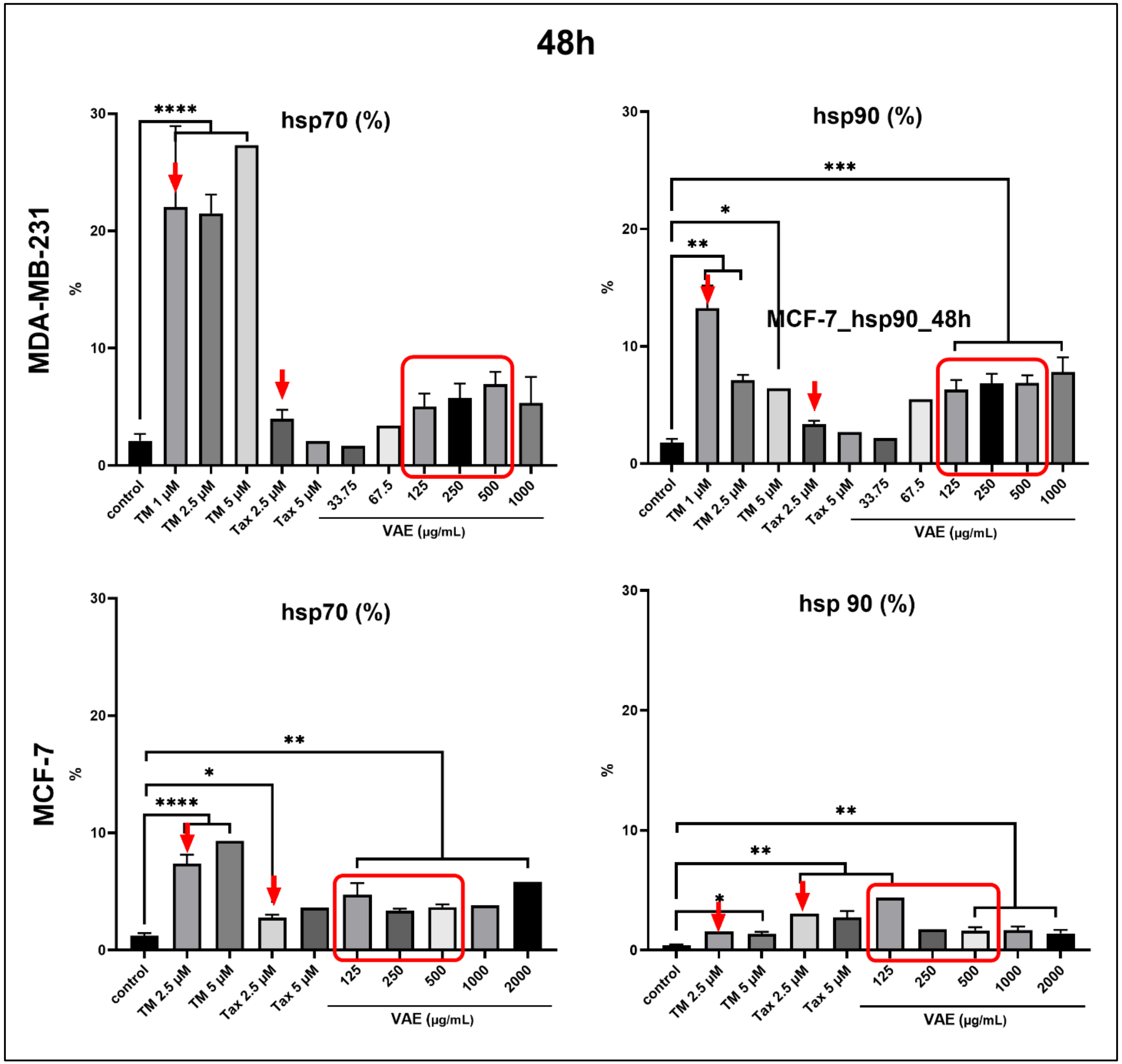

Suppl._3 Figure 4: Cell surface exposure of hsp70 and hap90 after treatment of MDA-MB-231 and MCF-7 cells, respectively for 48h with either tunicamycin (TM), taxol (Tax) or viscum album extract (VAE). The areas outlined in red, or marked with red arrows, indicate the conditions that were used for the final analysis.

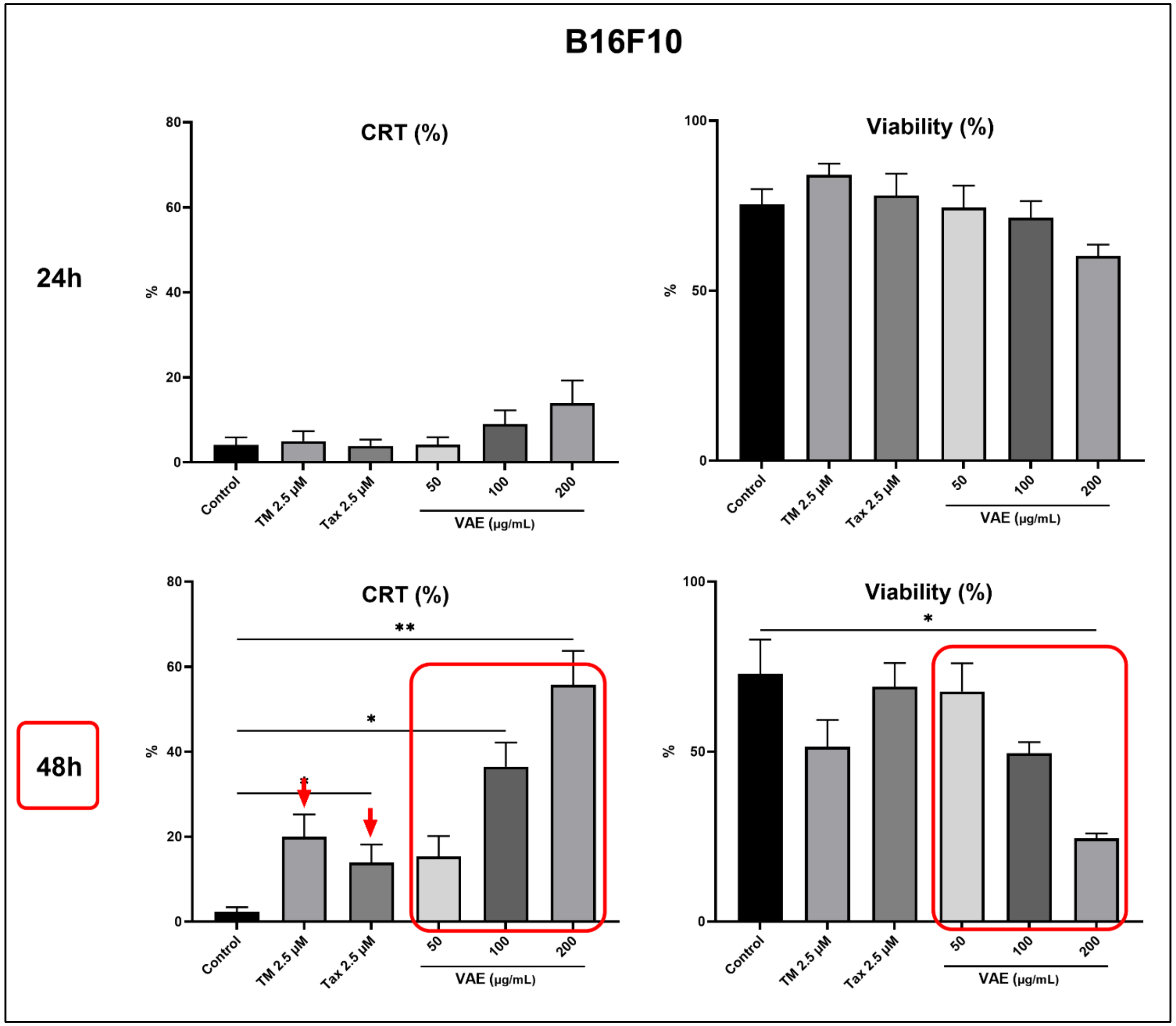

Suppl._3 Figure 5: Cell surface exposure of calreticulin (CRT) and viability after treatment of B16F10 cells for 24h or 48h with either tunicamycin (TM), taxol (Tax) or viscum album extract (VAE). The areas outlined in red, or marked with red arrows, indicate the conditions that were used for the final analysis.

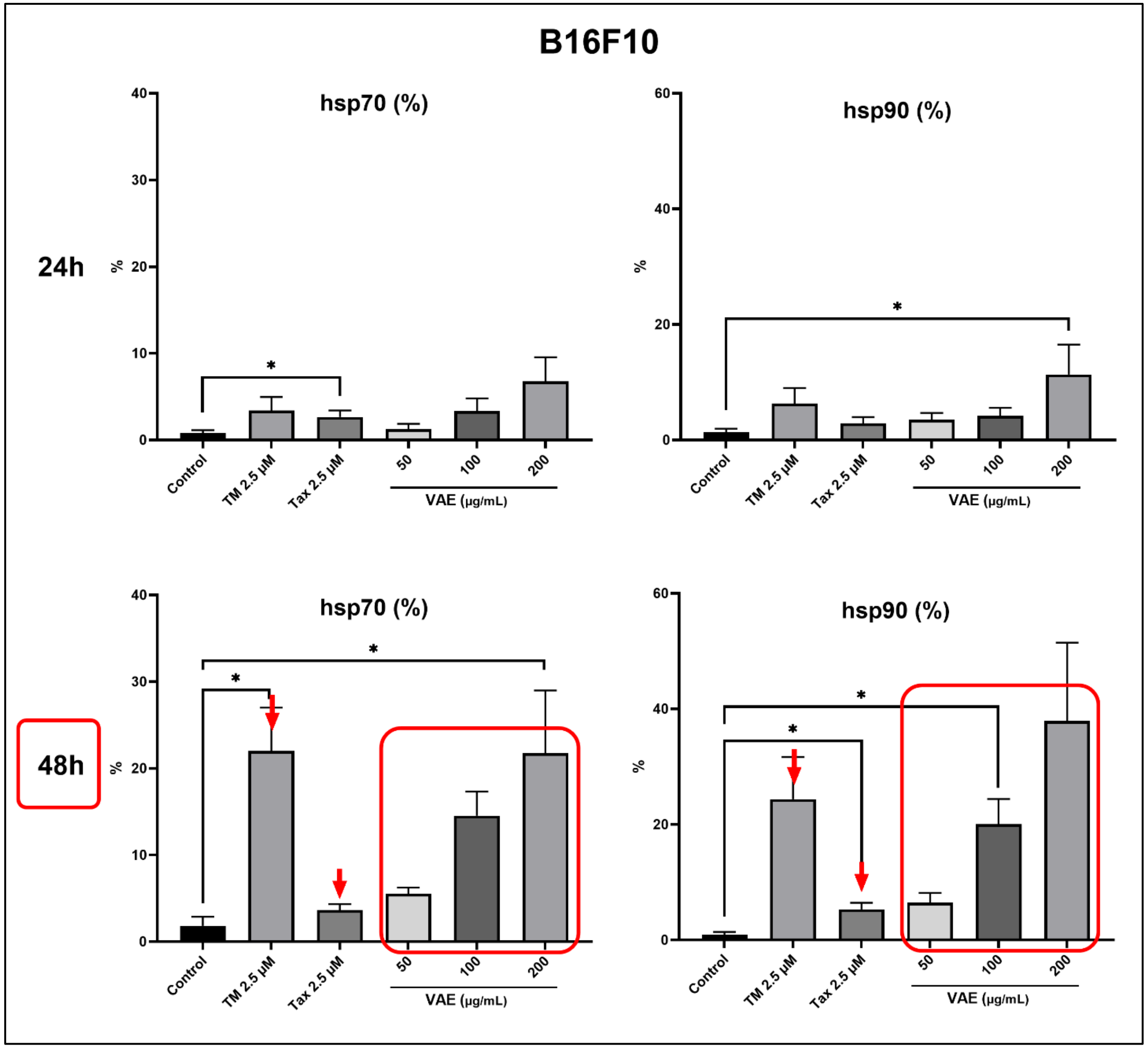

Suppl._3 Figure 6: Cell surface exposure of hsp70 and hap90 after treatment of B16F10 cells for 24h or 48h with either tunicamycin (TM), taxol (Tax) or viscum album extract (VAE). The areas outlined in red, or marked with red arrows, indicate the conditions that were used for the final analysis.

EIF2α dose response

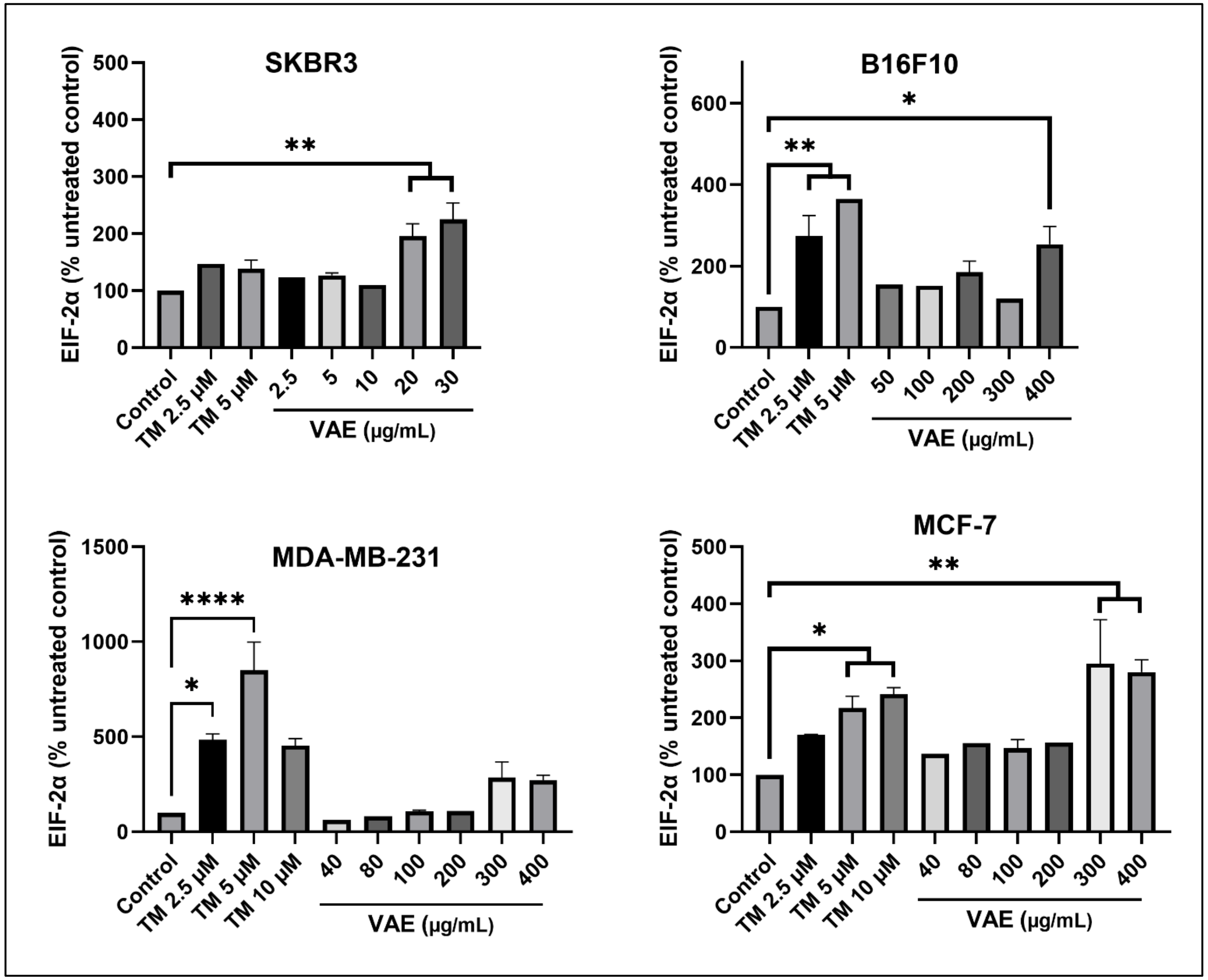

Suppl._3 Figure 7: The expression levels of p-eIF2α after indicated treatment for 24h were determined by stain free Western blot analysis.

Mitochondrial superoxide production

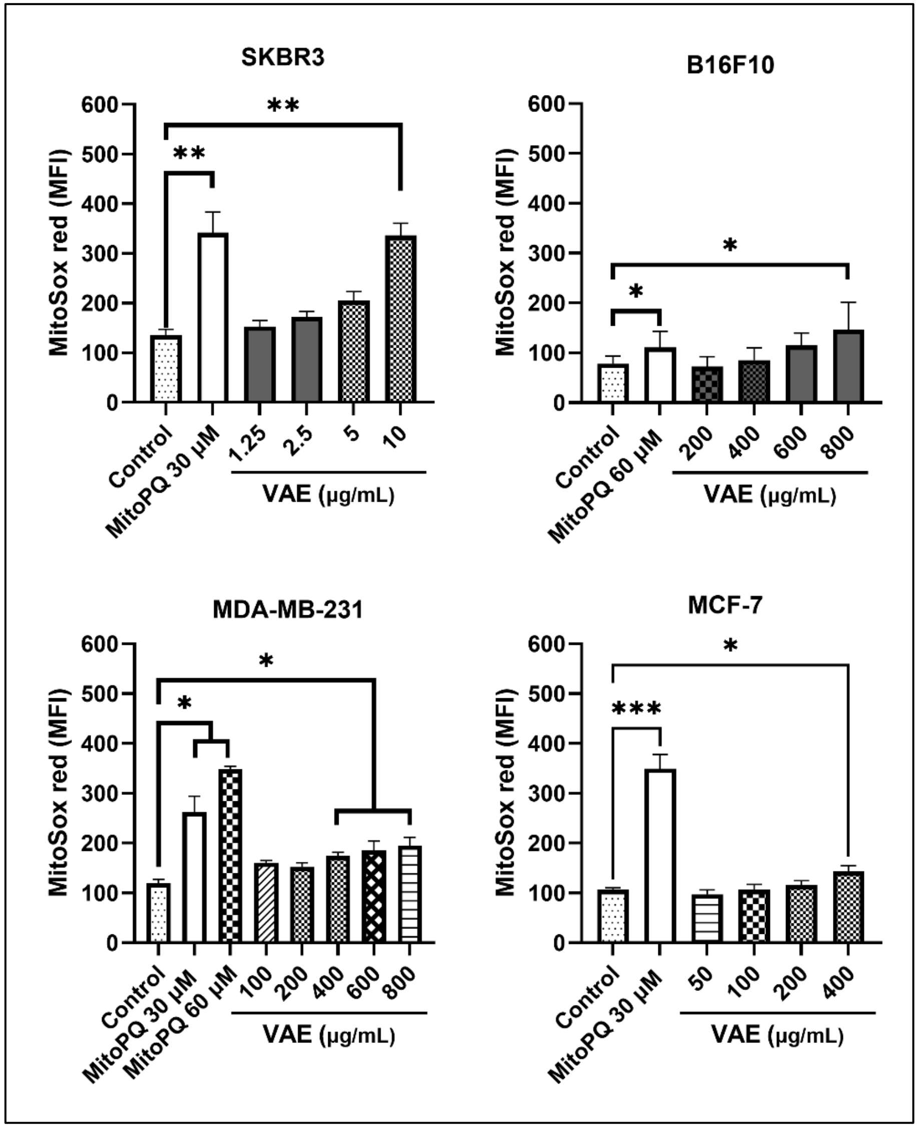

Suppl._3 Figure 8: Analysis of mitochondrial superoxide production. SKBR3, MDA-MB-231, MCF-7 and B16F10 cells were treated with either MitoPQ (positive control) or VAE for 24h, labeled with MitoSOX red Superoxide Indicator and analyzed by flow cytometry.
